# Viral infection of human neurons triggers strain-specific differences in host neuronal and viral transcriptomes

**DOI:** 10.1371/journal.ppat.1009441

**Published:** 2021-03-22

**Authors:** Colleen A. Mangold, Molly M. Rathbun, Daniel W. Renner, Chad V. Kuny, Moriah L. Szpara

**Affiliations:** 1 Departments of Biology, Biochemistry and Molecular Biology, Center for Infectious Disease Dynamics, and the Huck Institutes of the Life Sciences, Pennsylvania State University, University Park, Pennsylvania, United States of America; 2 Department of Entomology, College of Agricultural Sciences, Pennsylvania State University, University Park, Pennsylvania, United States of America; Oklahoma State Univeristy, UNITED STATES

## Abstract

Infection with herpes simplex virus 1 (HSV-1) occurs in over half the global population, causing recurrent orofacial and/or genital lesions. Individual strains of HSV-1 demonstrate differences in neurovirulence *in vivo*, suggesting that viral genetic differences may impact phenotype. Here differentiated SH-SY5Y human neuronal cells were infected with one of three HSV-1 strains known to differ in neurovirulence *in vivo*. Host and viral RNA were sequenced simultaneously, revealing strain-specific differences in both viral and host transcription in infected neurons. Neuronal morphology and immunofluorescence data highlight the pathological changes in neuronal cytoarchitecture induced by HSV-1 infection, which may reflect host transcriptional changes in pathways associated with adherens junctions, integrin signaling, and others. Comparison of viral protein levels in neurons and epithelial cells demonstrated that a number of differences were neuron-specific, suggesting that strain-to-strain variations in host and virus transcription are cell type-dependent. Together, these data demonstrate the importance of studying virus strain- and cell-type-specific factors that may contribute to neurovirulence *in vivo*, and highlight the specificity of HSV-1–host interactions.

## Author summary

Infection with herpes simplex virus 1 (HSV-1) affects a significant portion of the global population, and recent research has implicated persistent HSV-1 infection with the development of disease later in life, including neurodegenerative disease and cardiovascular disease. It is clear that individual strains of HSV-1 that exist within the circulating population exhibit specific genetic differences that affect their phenotypes in experimental settings. These differences in turn may contribute to the wide range of clinical outcomes observed between infected individuals. In this study, we sought to understand virus strain- and host-specific transcriptional changes during HSV-1 infection using an *in vitro* model of human neurons, a key cell type involved in HSV-1 persistence in humans. We show that different strains of HSV-1 demonstrate differences in viral gene expression and protein levels in infected human neuronal cells. These differences are not as pronounced in epithelial cells, suggesting that dissimilarities in viral gene expression and protein levels between strains may be cell-type specific. Infected neurons also exhibit unique transcriptional changes in response to specific HSV-1 strains, in pathways such as integrin signaling and remodeling of adherens junctions. Together, these data highlight the specificity of HSV-1 strain- and host-interactions, and the need to study the virus strain- and cell type-specific factors that contribute to HSV-1 pathogenesis.

## Introduction

Herpes simplex virus type 1 (HSV-1) is a human pathogen that affects over half the global population and causes recurrent epithelial lesions throughout an individual’s lifetime [1]. The HSV-1 lifecycle begins upon contact with mucosal surfaces, and it is in this niche where it actively replicates and can induce local lesion formation. The virus then enters local sensory nerve endings and traffics in a retrograde direction back to neuronal cell bodies in the peripheral nervous system (PNS). It is in this location where the virus enters into a latent, nonreplicative stage until later reactivation [2]. The ability of HSV-1 to infect and establish latency in neurons allows for lifelong infection, and can provide the virus with access to other sites such as the central nervous system (CNS). Recent research has implicated HSV-1 infection with the development of disease later in life, including Alzheimer’s disease [3–9]. It has been hypothesized that reactivating HSV-1 may travel from the site of latency in the trigeminal ganglia to areas of the brain known to be impacted by Alzheimer’s disease, resulting in subclinical inflammation and the formation of neuronal lesions [3, 10]. Similarly, reactivation of HSV-1 in autonomic nerves that innervate coronary arteries may introduce lytic virus to vascular endothelial cells, causing local injury and thrombosis [11] as well as potentially contributing to other cardiovascular disorders [11–15]. Despite these hypothesized connections between HSV-1 infection and disease later in life, the molecular mechanisms underlying neuronal responses to HSV-1 and the variability of these neuropathological effects due to differences between HSV-1 strains remains limited [9]. The study of both host and virus responses to infection in neurons is therefore critical to address these prevalent health concerns, and to elucidate host- and virus-specific factors that contribute to neurovirulence *in vivo*.

Previous work has sought to understand the effects of HSV-1 infection on neuronal transcription using a number of *in vitro* and *in vivo* neuronal models combined with microarray analysis of transcript expression, as reviewed in [16]. The neuronal models employed for these studies include primary rodent cells [17–22], immortalized murine neuroblastoma cell lines [23], and human teratocarcinoma cells [24]. These studies have provided a strong foundation of knowledge about common neuronal responses to HSV-1 infection across an array of cellular models and virus strains [16]. By applying RNA-sequencing methods to HSV-1 studies and using a human *in vitro* neuronal model, we can identify species-specific changes in host gene expression and simultaneously characterize viral gene expression, in a synchronized time course of infection. Several recent studies have used non-neuronal cell models (e.g., fibroblasts and other epithelial-like cells) with RNA-sequencing to study changes in host and virus transcription during HSV-1 infection [25–34]. These latter studies have significantly advanced our understanding of the effects of HSV-1 infection on host transcriptional processes during productive and quiescent infection of epithelial and fibroblast cells. However, in addition to their ongoing cell cycle, these cellular models for HSV infection lack the elaborate cellular architecture of mature neurons, and the expression of neuron-specific components such as synaptic proteins.

The ability of HSV-1 to travel from its site of latency in the peripheral ganglia to the CNS is hypothesized to be a crucial step in the development of disease later in life. Specific strains of HSV-1 exhibit differing abilities to reach the CNS from inoculation at peripheral sites in animal models (termed “neuroinvasion”) [35], and this phenotype is thought to contribute to their observed differences in overall neurovirulence, or ability to cause disease in the nervous system [36–38]. Evidence of disparities in neuroinvasion comes from a number of studies that combine the use of *in vivo* and *in vitro* models to assess the ability of different strains of HSV-1 to enter the nervous system and replicate in neurons [35, 39, 40]. Multiple aspects of the virus, host, and environment contribute to neurovirulence *in vivo*. These factors include the ability of the virus to replicate at epithelial sites of entry, to undergo axonal transport and replication in host neurons, and to evade the host immune system [35, 39, 40].

Three well-characterized strains of HSV-1 include F, KOS, and McKrae. Strains F and KOS were originally isolated from facial lesions whereas strain McKrae was isolated from a patient with herpes simplex keratitis [41–43]. Footpad inoculation of either strain F or KOS yielded lower mortality in comparison to clinical strains of HSV-1, suggesting that these clinical isolates were more neuroinvasive than either F or KOS [35]. Following ocular inoculation of either KOS or McKrae, McKrae demonstrated higher titers in the nervous system than KOS in two mouse strains [40]. This is true despite the fact that KOS replicated equally well if not better than McKrae over time in the murine corneal epithelium [40]. Strain KOS has a known point mutation in the Us9 gene, whose protein product plays key roles in neuronal infection [44–50]. Together these *in vivo* studies highlight strain-specific differences in the ability of HSV-1 to move within the nervous system as well as to cause pathology in neurons. Differences in the ability of any virus strain to reach the CNS may be due to host- or virus-specific factors, or a combination of the two. Strains F, KOS, and McKrae are all able to establish latency and spontaneously reactivate following high dose ocular inoculation in rabbits [51]. However, only McKrae can spontaneously reactivate via endogenous mechanisms in the rabbit, as well as via exogenous mechanisms such as adrenergic induction. This suggests that there may also be strain-specific differences in viral genes that play a role in reactivation [51]. Additionally, differences in amino acid sequences within HSV-1 glycoproteins involved in cell binding and entry have been proposed to contribute to the enhanced neuroinvasiveness observed in McKrae [52].

Comparative genomic and transcriptomic approaches have been used to identify factors associated with disease pathogenesis in several viruses, and have helped elucidate virus strain-dependent differences in host responses that may impact disease outcome [53–56]. To date, few studies have simultaneously analyzed both the host and virus transcriptomes in an effort to identify potential virus strain-dependent differences in gene transcription that may differentially impact host cell processes. In the study presented here, we infected mature neuronal cells derived by differentiation of human SH-SY5Y neuroblastoma cells [57] with one of three well-known laboratory strains of HSV-1 (F, KOS, and McKrae) [35, 40, 51] (**S1 Fig**). We then assessed neuronal and viral transcriptional responses to this productive infection at 12 and 24 hours post infection (hpi). Using RNA-sequencing and bioinformatics analyses, we compared the host and virus transcriptomes between the three viral strains and over time. We found that the virus strain used for infection had a significant impact on both viral- and host-gene expression. Additionally, we observed strain-dependent differences in viral protein levels in neurons that were less pronounced in non-neuronal cells (e.g. primate epithelial cells and human fibroblasts), suggesting that at least a portion of inter-strain differences in viral protein levels may be neuron-specific. Analysis of host-pathways differentially impacted by HSV-1 strain revealed strain-specific differences in adherens junction structure, integrin signaling, and others. The range of neuronal host responses to HSV-1 infection seen here highlight the need to examine virus-neuron interactions on a per-strain basis, rather than using epithelial cells as a universal model, or using a single virus strain to encompass all responses to a given viral species.

## Results

### Transcriptome analysis of HSV-1-infected human neuronal cells allows for the simultaneous assessment of both the virus and host

The present study sought to identify both host- and virus-specific factors that may contribute to previously observed strain-specific differences in HSV-1 neurovirulence *in vivo* [35, 40, 51]. To achieve this, we used a human neuronal cell model combined with RNA-sequencing to characterize strain-dependent differences in host cell responses as well as differences in viral gene transcription between HSV-1 strains and over time (**S1 Fig**). Immunofluorescence assays were performed to determine the number of purified virions of HSV-1 required to achieve synchronous infection in this neuronal model. Neuronal cultures were infected with a range of infectious doses and probed for HSV-1 protein at 14 hpi (**S2 Fig**). At lower concentrations, absence of anti-HSV-1 immunoreactivity was evident, particularly in strains F and KOS. However, at the highest infectious dose, all neuronal cell bodies for each strain had detectable viral protein consistent with productive replication (**S2 Fig**). This finding is in accordance with prior studies demonstrating that high multiplicities of infection are necessary to uniformly infect neurons with alphaherpesviruses [58–61]. For samples used in the RNA-sequencing analysis, differentiated SH-SY5Y human neuronal cells were infected with purified virions of HSV-1 strain KOS, F, or McKrae at 1.6e^7^ PFU/dish to ensure synchronous infection. Infected neuronal cells were harvested and total RNA extracted at 12 and 24 hpi. These time points have been shown previously to represent the midpoint and peak times of virus production by these neuronal cells [62]. After RNA-sequencing, differential gene expression of both the host and virus transcriptomes were compared relative to mock-infected neurons, between virus strains, and across time. Mock infections consistently showed little to no reads mapping to the HSV-1 transcriptome (average 94% host), and virus infections showed similar counts of reads mapping to each strain and at each time point (average 46% host, 43% HSV-1) (see **S1 Table** for sequence read statistics, and **S2 Table** for logCPM values of host transcripts).

### Neuronal responses to productive HSV-1 infection

**Fig 1:**
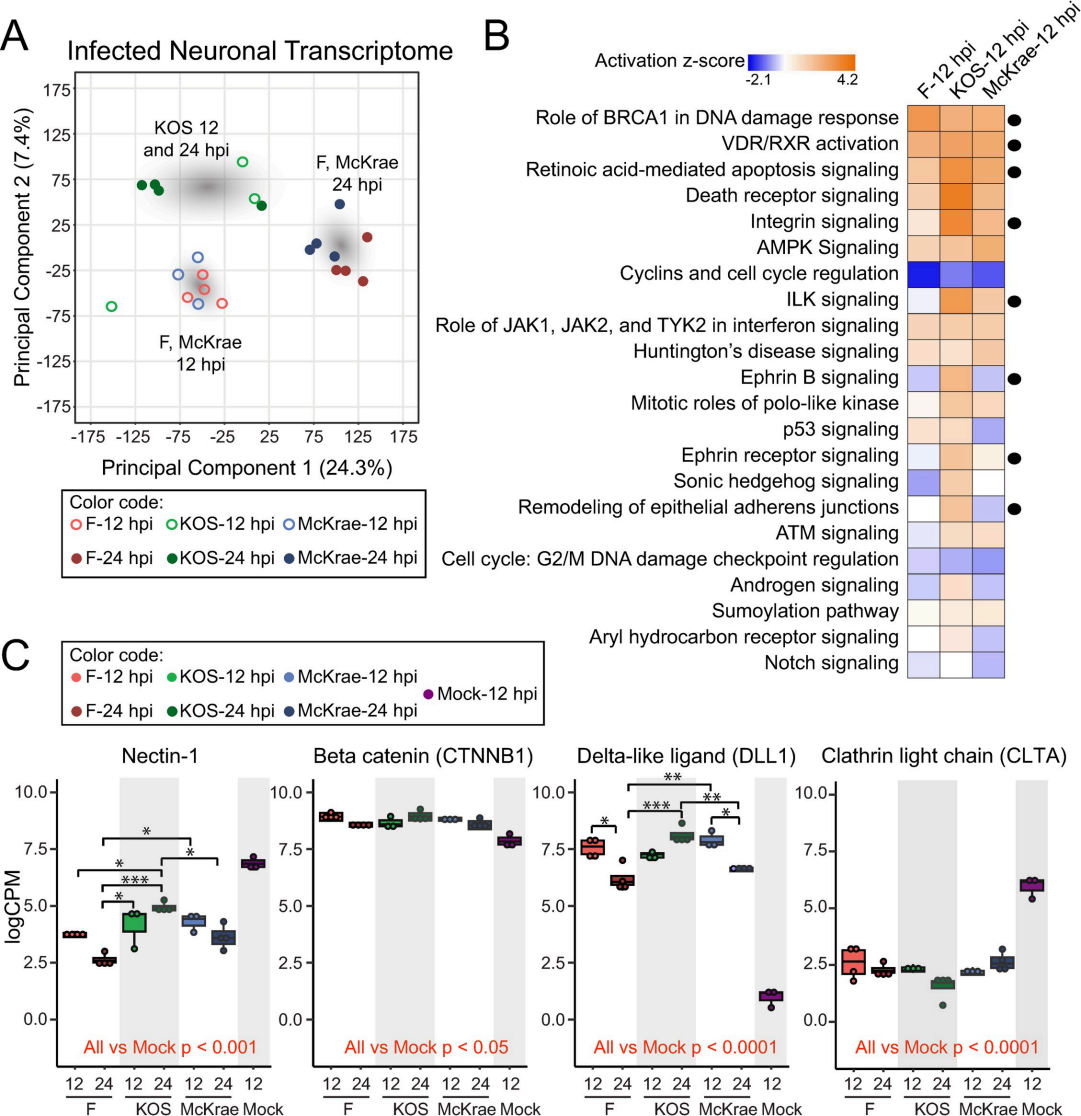
The host neuronal transcriptome demonstrates strain- and time-dependent differences in response to infection. **(A)** A plot of the principal component analysis (PCA) on the neuronal transcriptome at 12 and 24 hpi reveals three different groupings of samples. The host transcriptomes of neurons infected with either F or McKrae at 12 hpi and 24 hpi demonstrate similar patterns of transcript expression, while neurons infected with KOS cluster mostly together. Gray clouds have been added to illustrate these focal areas. **(B)** Host transcripts that were differentially expressed in neurons infected with either F, KOS, or McKrae versus Mock at 12 hpi (**S2 Table**) were used as input for three independent core analyses in Ingenuity Pathway Analysis, and the resulting pathways were then compared across strains. Shown here are the top 22 host pathways that were most significantly regulated in these comparisons (activation z-score = a score that predicts whether an upstream regulator in the pathway is activated or inactivated). These data suggest differential impacts of virus strain on multiple neuronal signaling pathways. Pathways discussed in the Results section are indicated with a black circle. All core pathways impacted by F, KOS, and McKrae infection are listed in **S3 Table** along with gene names. P-values and z-scores associated with the 22 pathways shown here are also included in **S3 Table**. **(C)** Transcripts of interest identified from the pathway analysis **(B)** were plotted individually to assess differential gene expression of specific transcripts. Transcripts were chosen based on their association with different pathways, including remodeling of epithelial adherens junctions (nectin-1, beta catenin [CTNNB1], and delta-like ligand [DLL1]) and endocytosis-related pathways (clathrin light chain A [CLTA]). Box plots show median, quartile ranges, and individual data points. A two-way ANOVA with post-hoc pairwise comparisons and a Bonferroni multiple testing correction was used to assess statistical significance. Comparisons versus mock-infected neurons are indicated in red font. *p < 0.05, **p < 0.001, ***p < 0.0001.

To determine whether there are any overt dissimilarities in host neuronal responses to productive infection with different strains of HSV-1, a principal components analysis (PCA) was performed to visualize patterns in gene expression between groups. With this approach, we can reduce the multitude of variability coming from each gene transcript count into two axes representing the most impactful composite effects that distinguish each test group. In doing so we detected the largest effect of group separation, or highest proportion of variance, between Principal Component 1 (PC1, 24.3%) and Principal Component 2 (PC2, 7.4%). Differences in host transcription in response to infection were dependent on virus strain in addition to the duration of infection (**Fig 1A**). The transcriptional profiles of neurons infected with KOS at 12 and 24 hpi clustered closely to each other (**Fig 1A**), and were separated from the profiles of neurons infected with F or McKrae at either timepoint. In contrast, host transcriptional changes due to infection with F and McKrae clustered together at both 12 and 24 hpi (**Fig 1A**). Each of these clusters was manually highlighted in Fig 1A to depict the observed differences.

In order to identify host pathways that were impacted by each strain, a core pathway analysis was performed on all host transcripts identified as differentially expressed in virus-infected versus mock-infected neurons at 12 hpi for HSV-1 F, KOS, and McKrae respectively (see **S2 Table** for list of all differentially expressed host transcripts). All host pathways impacted by F, KOS, and McKrae infection are listed in **S3 Table** along with the specific genes that were differentially expressed in each pathway. Pathways that were identified as activated (positive z-score) or deactivated (negative z-score) following infection (p < 0.05 in at least one comparison) are shown in **Fig 1B** and listed in **S3 Table,** along with respective -log(p-values) and z-scores. Many host pathways and genes identified as differentially expressed in the current analysis corroborate previous findings [16]. Host processes that were most highly regulated (increased or decreased) by infection regardless of strain included the role of breast cancer type 1 susceptibility protein (BRCA1) in DNA damage response, vitamin D receptor/retinoid X receptor (VDR/RXR) activation, and retinoic acid mediated apoptosis signaling (**Fig 1B**). Other pathways that demonstrated differential regulation by infection included integrin-linked kinase (ILK), integrin-, ephrin B-, and ephrin receptor-signaling as well as remodeling of epithelial adherens junctions. Specifically, KOS-infected neurons demonstrated higher activation of the latter pathways in comparison to neuronal cells infected with either HSV-1 F or McKrae. Several of these pathways, such as integrin-, ephrin B-, and ephrin receptor-signaling as well as adherens junction structure are involved in synaptic and cytoskeletal structure [63]. These data suggest that KOS infection induces differential responses in synaptic and cytoskeletal morphology that may impact its intracellular transport, cell-to-cell transmission, and potentially its neurovirulence *in vivo*.

Genes identified within pathways of interest were individually plotted (**Fig 1C**). In particular, nectin-1, beta-catenin (CTNNB1, or catenin beta 1), and delta-like canonical Notch ligand 1 (DLL1) were chosen from the remodeling of epithelial adherens junctions pathway, and clathrin light chain A (CLTA) was chosen from endocytosis-related pathways. Specific transcripts were chosen based on raw expression levels (i.e., the gene transcript that demonstrated the highest level of expression was chosen as the ‘main’ transcript and plotted in **Fig 1C**) (see **S2 Table** for list of all differentially expressed host transcripts). Some transcripts of interest showed differential expression in all viral-infected groups versus mock-infected neurons only (e.g. beta-catenin and clathrin light chain), while others also displayed significant differences between viral strains (e.g. nectin-1 and delta-like DLL1). Of note, alterations in host gene expression did not occur in one direction only; for example, nectin-1 decreased in expression in all infected neurons as compared to mock, while delta-like DLL1 expression increased in all infected neurons (**Fig 1C**).

### HSV-1 strains KOS, F, and McKrae demonstrate differential viral gene expression patterns in neurons

**Fig 2:**
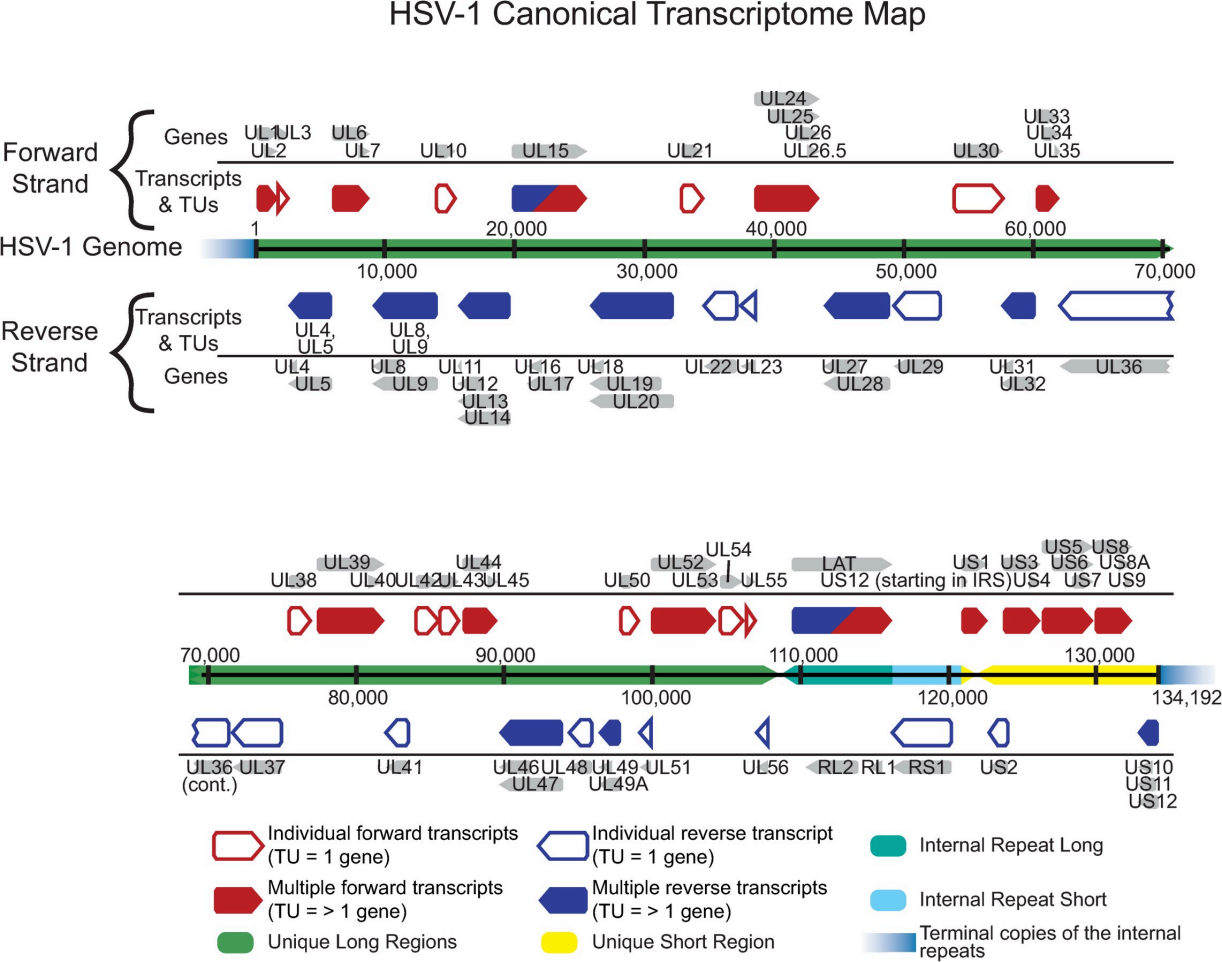
HSV-1 transcriptome map. The HSV-1 transcriptome includes both single-gene transcripts and 3’ co-terminal, overlapping transcriptional units (TUs), which are assigned to shared TU’s in order to analyze differences in viral gene expression between HSV-1 strains F, KOS, and McKrae. Red boxes indicate transcripts encoded on the forward strand while blue boxes indicate transcripts encoded on the reverse strand. Grey arrows indicate individual canonical genes encoded by the HSV-1 reference genome (JN555585, strain 17). Where there is overlap, genes are grouped together into a single TU (red/blue-filled annotations) while in areas without overlap, genes are assigned as individual transcripts (red/blue-outlined annotations). HSV-1 gene assignments within each TU are noted. Spatial coordinates of the 152 kb HSV-1 genome indicate the classic nomenclature of unique long (green bars) and unique short (yellow bar) regions, which are flanked by internal and terminal inverted repeats (aqua bars). Terminal repeats (fading blue bars) are not included in full in order to devote more space to unique annotations.

**Fig 3:**
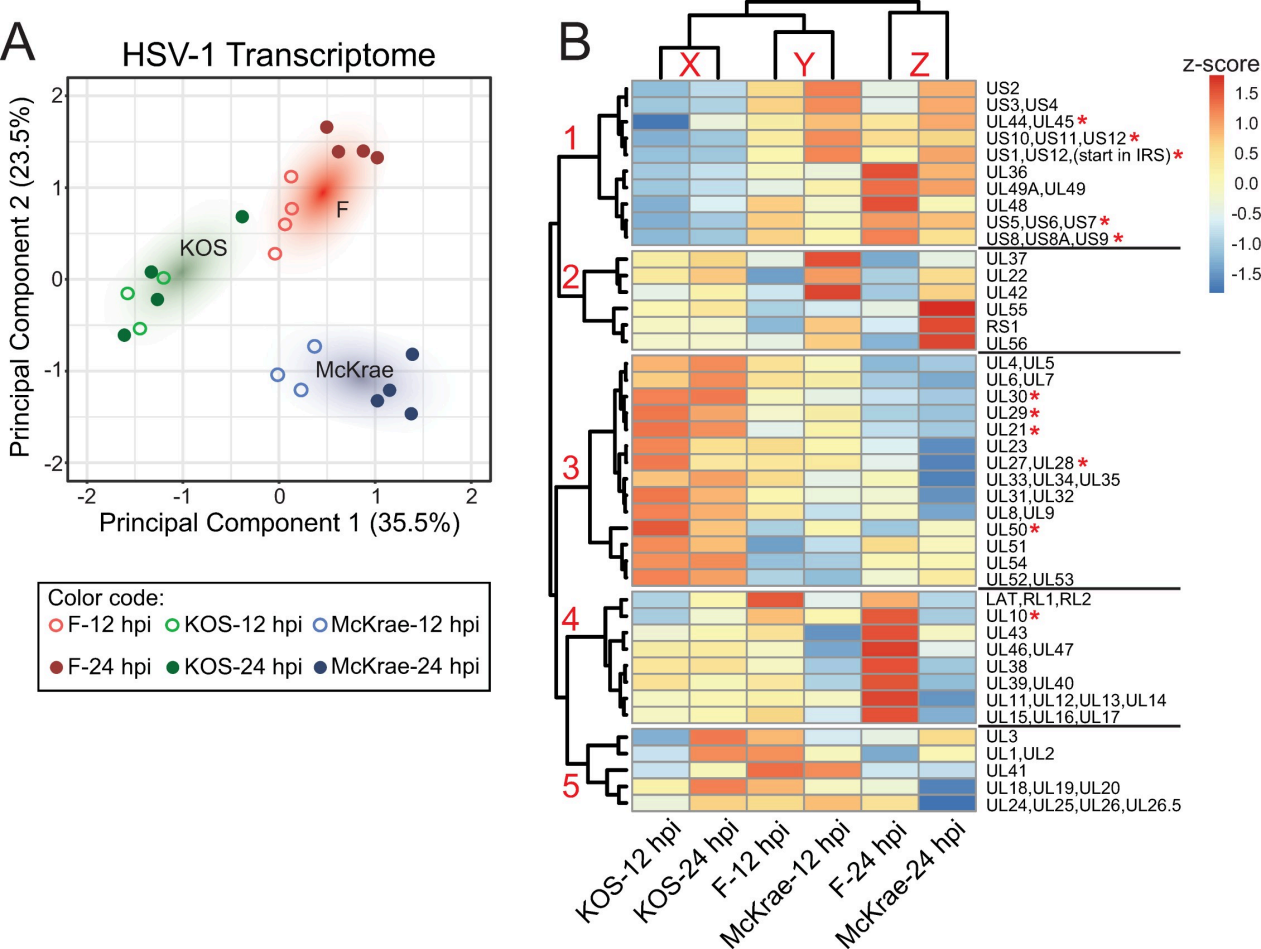
Comparison of viral transcriptomes reveals distinct differences between strains in viral gene expression during neuronal infection. **(A)** A plot of the principal components analysis (PCA) of viral transcriptional unit (TU) expression in infected SH-SY5Y neurons demonstrates the separation of samples based on virus strain. A colored cloud has been added to highlight the focal areas of specific virus strains (KOS = green, F = red, McKrae = blue). Differences between HSV-1 strains account for most of the viral transcriptome variance. Minor separation based on time (hpi) is evident in strains F and McKrae. In contrast, the transcriptomes of neurons infected with strain KOS for either 12 or 24 hpi cluster together, suggesting a slower progression of change in this strain. **(B)** Heatmap analysis of virus TU expression shows five clusters of TUs (red numbers on dendrogram at left). Data are normalized by row by z-score with hierarchical clustering (Pearson correlation). The two largest clusters (labelled 1 and 3) depict TUs with either lowest, or highest, expression by HSV-1 KOS strain. Another two clusters show F (cluster 4) or McKrae (cluster 2) as having the highest or lowest expression. In cluster 5, the highest and lowest TU expression depends on both strain and time post-infection. As seen in the PCA plot at left, overall patterns in KOS cluster together regardless of time (vertical dendrogram label X), whereas F and McKrae are interleaved based on time point (vertical dendrogram, Y, Z). TU’s with statistically significant differences in expression between groups are marked with a red asterisk (see Fig 4 for details).

The HSV-1 reference genome contains at least 76 canonical open reading frames, of which 49 are 3’ co-terminal [34, 64, 65]. Each set of overlapping, co-terminal genes was grouped together and counted as one transcriptional unit (TU) [30] to avoid ambiguity in mapping of reads to these areas of transcriptional overlap (**Fig 2, S4 Table**). Comparison of viral gene expression between infected groups and over time indicated that each virus strain exhibited distinct expression patterns of its TUs (**Fig 3**). Additionally, it was evident through our PCA (principal components PC1 = 35.5%, PC2 = 23.5%) that virus strain had a greater impact on viral gene expression than time post infection (**Fig 3A**). Differences in viral gene expression observed between strains were maintained across time, as evidenced by grouping of the 12 and 24 hpi time points within strain (**Fig 3A**). With the exception of KOS-infected neurons, the 12 and 24 hpi data formed sub-groups suggesting that there are changes in virus TU expression that are specific to these time points. These observations were manually highlighted in **Fig 3A**.

Given the differences in viral transcription that are evident between strains, we tested for differences in productive viral replication in this neuronal model. Viral titers were quantitated at 0, 6, 12, 24, and 48 hpi and compared between HSV-1 strains F, KOS, and McKrae (**S3 Fig**). A two-way analysis of variance (ANOVA) revealed a significant main effect of time post infection on viral titer (p<0.0001) with no significant main effect of virus strain. However, a significant interaction was identified between strain and time that impacts viral titer (p<0.0001). Post hoc analysis with a Bonferroni multiple testing correction (MTC) revealed no significant differences in viral titer evident between strains at 0, 6, and 12 hpi. At 24 hpi, strain F demonstrated a higher titer than McKrae (p = 0.009), and at 48 hpi strain F demonstrated a lower titer than both McKrae and KOS (p<0.001). Together, these results demonstrate that F, KOS, and McKrae exhibit similar growth kinetics at the early infection time points in our transcriptome analysis.

To explore these groups further, we looked for broad trends across viral TUs using hierarchical clustering of TU expression levels (logCPM) normalized by z-score (**Fig 3B**). Five major clusters resulted from this analysis. Clusters one and three (as labeled in **Fig 3B**) contained the majority of TUs and were distinguishable by TUs with the highest overall expression in F- or McKrae-infected (cluster 1) or KOS-infected cells (cluster 3), respectively. Clusters two and four were similar in that they showed relatively moderate KOS-derived TU expression, and alternated whether the highest or lowest TU expression came from F- or McKrae-infected cells. Finally, TUs in cluster five showed a more varied pattern of expression. Virus gene families (**S4 Table**) were distributed across these clusters, further supporting a non-uniform differential expression pattern to distinguish each strain. Of note, KOS TU expression patterns at 12 and 24 hpi (vertical cluster X) were more similar to each other than to F and McKrae at either 12 (vertical cluster Y) or 24 hpi (vertical cluster Z) (**Fig 3B**, vertical dendrograms), in keeping with the patterns of host responses to these strains (see **Fig 1A**). Additionally, KOS TU expression at 24 hpi was more similar to that exhibited by F and McKrae at 12 hpi than to F or McKrae at 24 hpi. These data highlight the viral transcriptional differences that exist between HSV-1 strains in neurons.

**Fig 4:**
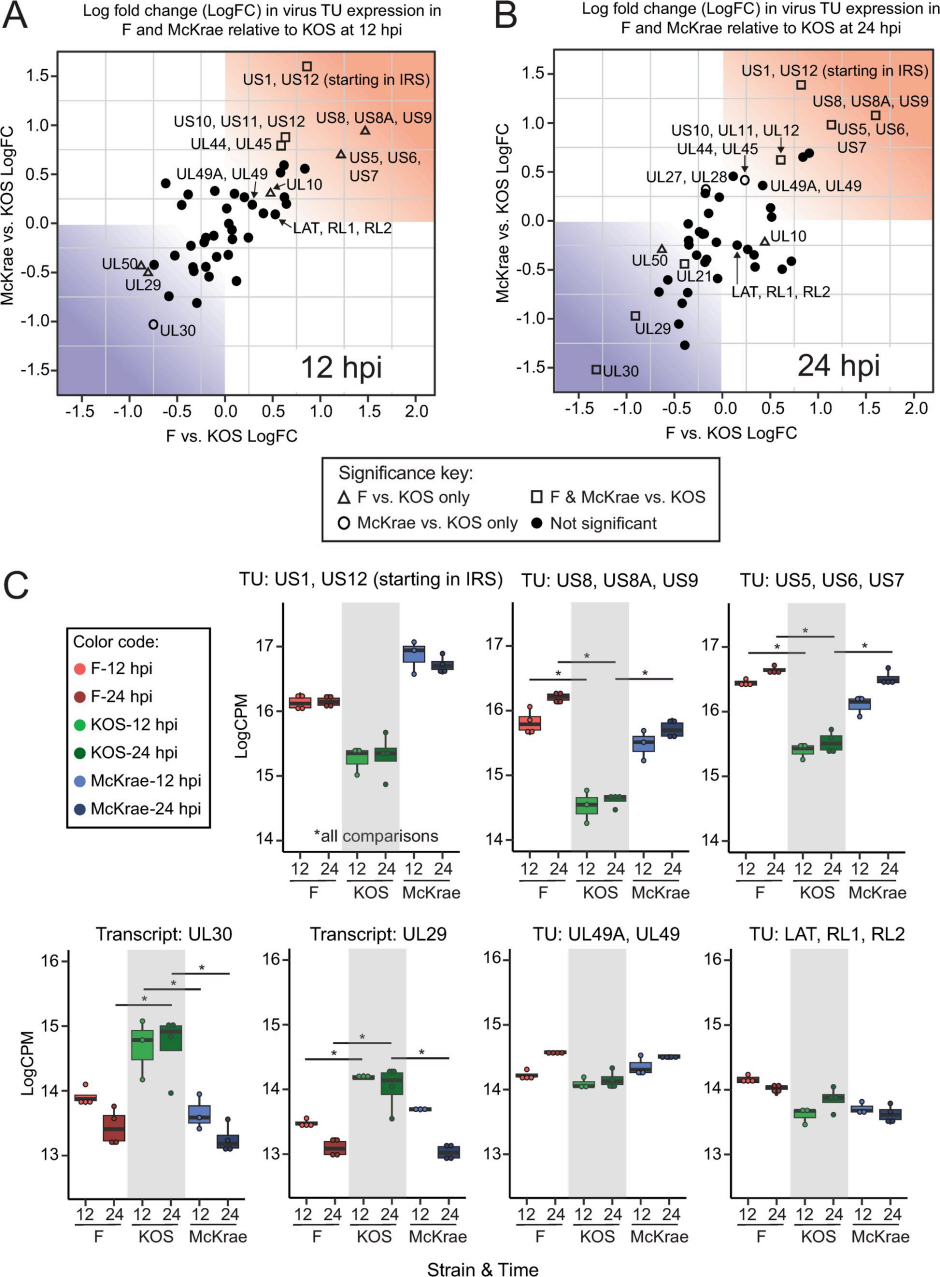
Visualization of individual viral transcripts and transcriptional units (TUs) highlights specific genes that exhibit differences in expression between strains. Scatterplots graphically depict the log_2_fold change (logFC) of HSV-1 strains F vs. KOS (x-axis) relative to strains McKrae vs. KOS (y-axis). These data are plotted separately for (**A**) 12 hpi or (**B**) 24 hpi. The upper right quadrant of each graph (shaded in red) depicts TUs that show higher expression in strains F or McKrae relative to KOS, while the bottom right quadrant (shaded in blue) shows those TUs that are lower in expression in F or McKrae compared to KOS. HSV-1 strains McKrae and F have more similar transcriptional profiles to each other than to strain KOS. Using a quasi-likelihood F-test, 11 TUs were found to be statistically significant (p<0.05, FDR<0.05) in two or more comparisons. These are highlighted as open triangles, squares, or circles (see key). (**C**) Examples of each class of TU are individually plotted to highlight particular differences in transcription between strains. The top row shows TUs from the red quadrant, which have higher expression in strains McKrae and/or F than KOS. The bottom left plots show TUs from the blue quadrant (UL30, UL29), where expression in KOS is higher than in F and/or McKrae strains. Finally the bottom right plots illustrate TUs for which no statistical significance in expression levels between strains was detected. Box plots show median, quartile ranges, and individual data points.

To investigate the most pronounced differences in viral gene transcription between strains, we fit a generalized linear model to the viral TU expression data, and contrasted groups by strain and time using a quasi-likelihood F-test. Of the 43 TUs, 11 were statistically significant (p<0.05, FDR<0.05) in at least two comparisons, and revealed a log fold change (logFC) ranging from 0.4–1.6 logCPM (**Fig 4**, see also **Fig 3B** and **S4 Table**). Comparisons that contrasted KOS vs. F or McKrae showed the highest number of differentially expressed TUs, which became more pronounced at 24 hpi. Contrasts between time points of the same strain showed almost no differential expression of TUs (**S4 Table**). Notably, these differences in gene expression consisted largely of TUs involved in DNA replication and DNA binding, as well as transcriptional regulation, and several glycoproteins (**Fig 4**, **S4 Table**). In particular, the TU containing the host and viral transcriptional regulator ICP22 (TU: US1, US12, start in IRS) showed less expression in KOS-infected cells than F- or McKrae-infections (p<0.00004, FDR<0.0009) (**Fig 4B**). Of the significant TUs, four out of five encoding a glycoprotein were expressed at a lower level in KOS-infected cells than in cells infected with F or McKrae. These include the axon-transport and viral egress associated TU: US8, US8A, US9, in which US8 encodes glycoprotein gE (p<0.001, FDR<0.007) (**Fig 4B**), and the viral fusion-associated glycoproteins gJ, gD, and gI that are encoded by TU: US5, US6, US7 (p<0.004, FDR<0.02).

A subset of viral TUs showed significantly higher expression in KOS-infected cells than in cells infected with F or McKrae (**Fig 4B**). Specifically, UL30, the virus-encoded DNA polymerase, demonstrated highest expression in KOS-infected neurons, and comparable expression between F- and McKrae-infected neurons (p<0.0002, FDR<0.002). Additionally, UL29, which encodes the integral DNA binding protein ICP8, was more highly expressed in KOS-infected neurons than in those infected with either F or McKrae (p<0.002, FDR<0.02). These differences either became significant at the 24-hour timepoint, or were already present at the 12-hour timepoint (**S4 Table)**. Together, these data demonstrate that the viral transcriptome of each strain is slightly different, with a shared transcriptional program playing out with different timing and/or levels of each viral transcript. The differential gene expression of specific TUs may underlie the previously-observed differences in neurovirulence *in vivo* between these virus strains.

### Morphological impacts of HSV-1 infection on human neurons

**Fig 5:**
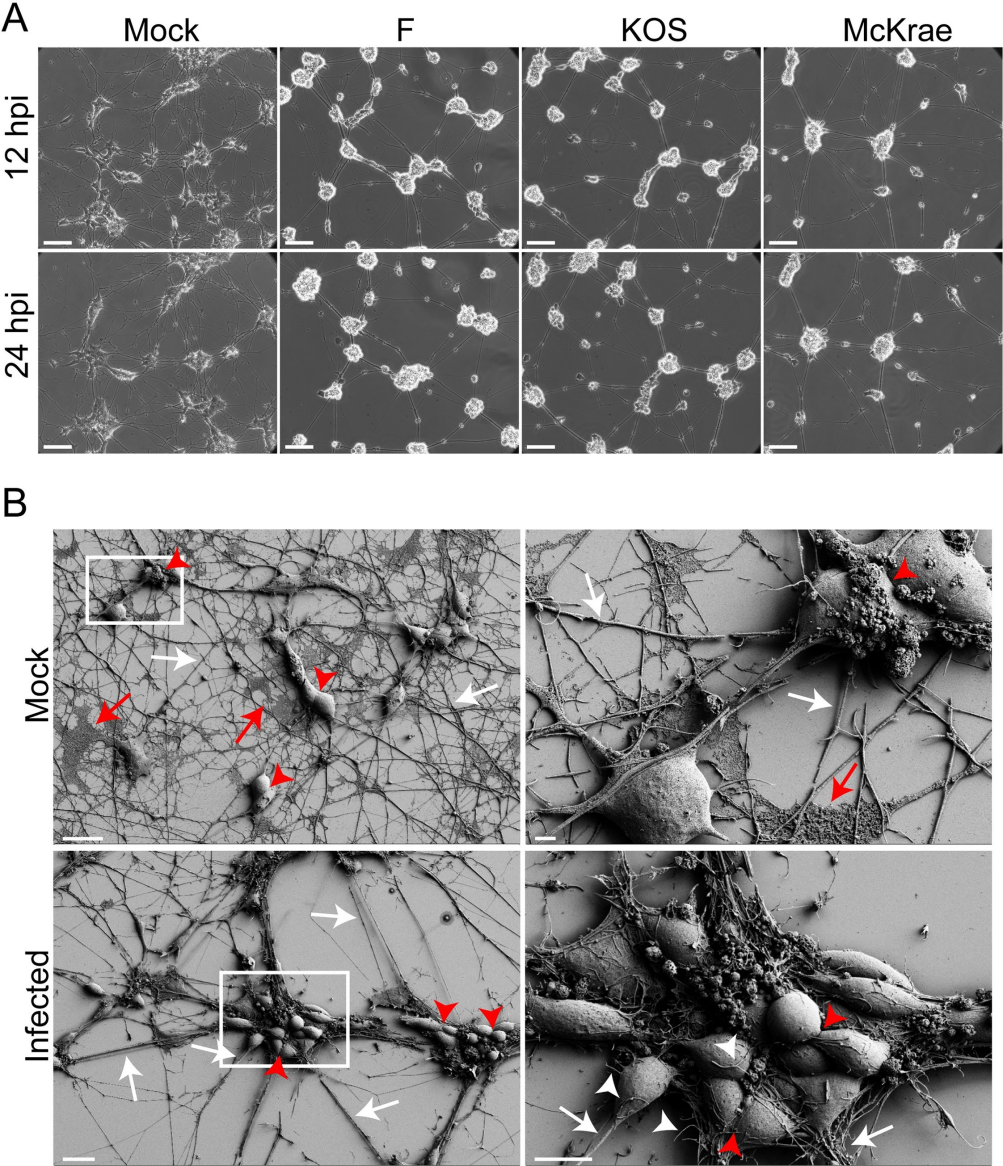
Brightfield and scanning electron microscopic (SEM) analysis of differentiated SH-SY5Y neuroblastoma cells reveals morphological and phenotypic changes that occur in neurons following infection with HSV-1. **A).** Brightfield microscopy illuminates mock-infected neurons as small clusters of individual cell bodies, with extensive networks of neurites projecting outward to surrounding cell clusters. Minimal movement of cell bodies and neurites over the course of 24 hours was evident. All infected neurons regardless of strain demonstrated cell body rounding, clustering of cell bodies, and detachment and fasciculation of neurites. Images shown here are representative of a time series shown in **S1–S4 Movies**. Scale bars = 100 μm. Images were acquired at 20X magnification once every hour for 24 hours (see **S4 Fig** for inverted images, which highlight the reduced adhesion and increased fasciculation of neurites). **B)** SEM analysis of infected SH-SY5Y cells reveals more detail of the changes in neuronal morphology following HSV-1 infection. Mock-infected neurons possess numerous long and thin neurites that appear to contact neighboring cells (white arrows). The neuronal cell bodies are oblong and cell-to-cell contacts are evident (red arrowheads), as is extracellular matrix (red arrows). Scale bar = 20 μm. The right-hand panel shows a 5,770X magnification of the inset marked in the left-hand panel. Scale bar = 2 μm. Differentiated SH-SY5Y neuroblastoma cells were infected with McKrae at an MOI of 10. At 6 hpi, neuronal processes fasciculate together (white arrows), extracellular matrix is less evident, cell bodies round up, and cell boundaries are more apparent (red arrowheads). In addition, numerous short, thin filipodia extend from cell bodies (white arrowheads). Scale bar = 20 μm. Right-hand panel shows a 3,320X magnification of inset marked in the left-hand panel. Scale bar = 10 μm.

The host pathway analysis above revealed potential virus strain-specific differences in host-cell synaptic remodeling, neuronal connectivity, and cell cytoarchitecture during productive infection. We thus sought to confirm broad changes in neuronal morphology following HSV-1 infection using microscopy. To observe whether alterations in neuronal morphology reflected the host processes identified in the pathway analysis (**Fig 1B**), we performed time-lapse microscopy and scanning electron microscopy (SEM) of these human neuronal cells over the course of infection. Regardless of the virus strain used, neurons infected with HSV-1 F, KOS, or McKrae began to round up and clustered together by 12 hpi (**Fig 5A**; see **S1–S4 Movies** for full image series, and **S4 Fig** for inverted images). Additionally, the multiple long neurites emerging from each neuronal cell body began to fasciculate together after infection, and neuron-to-neuron contacts were also visibly impacted. These data correlate well with the host transcriptional pathway changes observed above (**Fig 1B**).

To obtain a higher resolution view of changes in cell-to-cell contacts and morphology in this setting, we performed SEM on HSV-1-infected neurons. Under normal conditions, differentiated SH-SY5Y neurons formed small clusters of neuronal cell bodies with well-defined cell-to-cell contacts (**Fig 5B**). Neurites projecting from cell bodies were long and distinct, and appeared to contact neighboring neurons. Additionally, extracellular matrix material was apparent in areas underlying neurites and neuronal cell bodies. In contrast, neurons infected with HSV-1 McKrae for just 6 hours showed a distinct increase in clustering of neuronal cell bodies (**Fig 5B**). In addition, cell borders changed in infected neurons, as neurons rounded up and points of contact between cell bodies became smaller. Neurites fasciculated together, while areas of extracellular matrix became less evident. Additionally, small filopodia-like projections formed diffusely across neuronal cell bodies (**Fig 5B**, white arrowheads). This observation agrees with previously published data, which demonstrated that increased formation of F-actin-based dendritic filopodia may aid in early infection of neurons [66]. Together, these morphological data supported the findings from the host pathway analysis, in that both analyses indicated alterations in cell-to-cell contacts and neuronal cytoarchitecture as a consequence of HSV-1 infection.

### Assessment of viral protein levels

**Fig 6:**
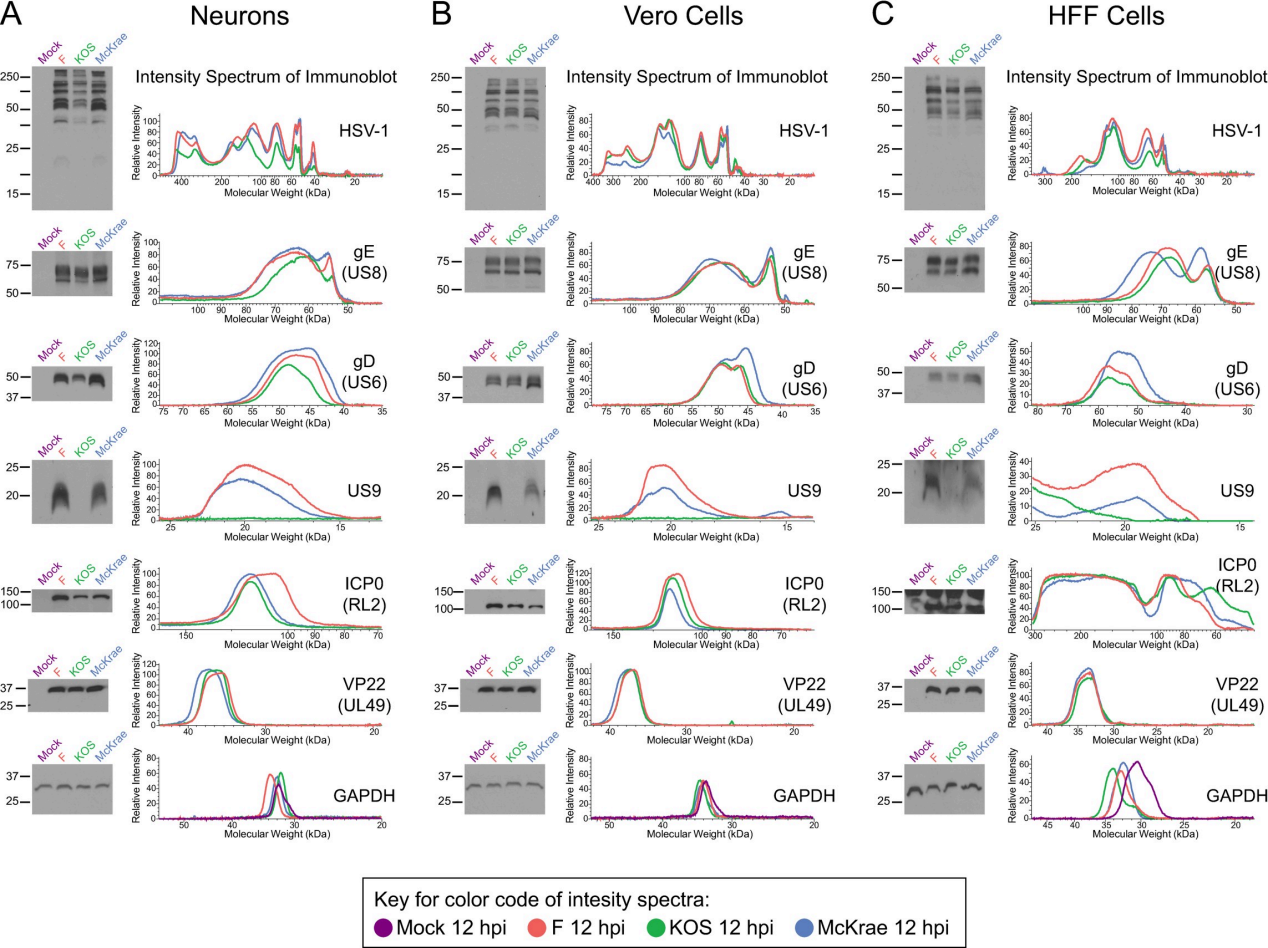
Different viral protein levels between strains demonstrates cell type specificity. **(A)** In neurons, immunoblot analysis of total HSV-1 protein, gE, gD, and US9 demonstrates different viral protein levels between strains at 12 hpi. HSV-1 strain KOS exhibited overall lower levels of total viral protein as well as less gD (see also **S5 Fig**). Additionally, marginally lower gE levels and a complete lack of US9 expression were observed in KOS-infected neurons. In contrast, VP22 protein levels were relatively consistent expression between strains. Subtle differences in total protein, ICP0, gE, gD, and US9 levels between strains were particularly evident in the corresponding intensity spectra. In contrast to the neuronal infections depicted in **(A)**, Vero epithelial cell lysates did not reveal lower levels of total viral protein, gE, or gD in KOS-infected cells **(B)**. Due to the known point mutation in US9 [44–46], this protein is not detected in Vero cells either. Consistent with neurons, no differences in VP22 levels between strains were evident in Vero cells and subtle differences such as higher levels of US9 and ICP0 in strain F persisted in both cell types. **(C)** Western blots in primary human fibroblasts (HFFs) demonstrate similar trends in viral protein levels as observed in neurons, although to a marginally lesser degree. In HFFs the immunoreactivity of a high molecular weight band at roughly 150 kDa was detected in all lanes, including mock-infected cells, and likely indicates non-specific binding of anti-ICP0 antibody to an HFF-specific cellular protein. Blots shown are representative of 3–4 biological replicates, each prepared and immunoblotted independently. All blots depicted in **A**–**C** come from a single one of these biological replicates.

In order to explore how the observed changes in host and viral transcription are translated into infection-related changes in neuronal morphology, we performed targeted Western blots to explore several viral proteins in greater detail. At 12 hpi, neurons infected with HSV-1 KOS exhibited lower total viral protein levels than neurons infected with either F or McKrae (**Fig 6A**). In contrast, in Vero cells infected with the same three strains for 6 hours, equivalent levels of total viral protein were observed (**Fig 6B**). The levels of total viral protein in primary human foreskin fibroblasts (HFFs) at 6 hpi were not as equivalent across HSV-1 strains as observed in Vero cells (**Figs 6C** and **S5A**), and viral protein levels in KOS-infected HFFs were marginally lower than in cells infected with either F or McKrae. At an RNA level, the TU encoding US8, US8A, US9 demonstrated significantly less expression in KOS-infected neurons than neurons infected with either F (1.6 log_2_-fold lower) or McKrae (1.1 log_2_-fold lower) (**Fig 4B**). To examine these differences at the protein level, US8 (gE) and US9 were analyzed by Western blot. KOS-infected neurons, HFF, and Vero cells demonstrated no discernable US9 protein (**Fig 6**), which is consistent with a known point mutation in the KOS gene encoding US9 [44–46]. Consistent with what was observed at the RNA level (**Fig 4B**), the level of US9 protein was higher in strain F than strain McKrae. While all infected neurons showed gE protein at 12 hpi regardless of strain, neurons infected with KOS reproducibly exhibited less overall gE immunoreactivity, as well as reduced expression of the lower band in particular (**Figs 6A** and **S5A**). The upper, 75–80 kDa band has been hypothesized to represent mature fully glycosylated gE, while the lower 65 kDa band is thought to represent the cleaved, partially-glycosylated precursor of gE [67, 68]. This suggests that in neurons, KOS may produce less precursor gE than the other two strains, and/or these may be polar effects of the US9 mutation in KOS. Once again, strain-specific differences in total gE protein levels (both the upper and lower bands) were not observed in infected Vero cells (**Fig 6B**). In human fibroblasts (HFFs), marginal differences in the levels of gE were also observed between strains (**Figs 6C** and **S5A**); however, in contrast to neurons, the lower band appeared to demonstrate similar levels across all three strains. As predicted by the transcriptional analysis (**Fig 4B**), the viral glycoprotein gD (US6) also demonstrated lower protein levels in KOS-infected neurons than those infected with strains F or McKrae (**Figs 6A** and **S5A**). The virus strain-specific difference observed in neurons was not equally detected in Vero cells, as KOS-infected Vero cells had equivalent levels of gD protein relative to F and McKrae. However, McKrae-infected Vero cells demonstrated higher levels of the lower molecular weight band (**Figs 6B** and **S5A**). In human fibroblasts, the strain-specific patterns of gD protein levels mirrored those observed in neurons (**Figs 6B** and **S5**). These data emphasize the value of examining HSV-1 strain-specific differences in different cell types. Non-neuronal cells such as Vero and HFF cells are not necessarily predictive of differences in viral protein levels seen in neurons.

Several TUs did not show significant differences in gene expression between strains and over time in the neuronal transcriptome analysis. RNA expression of the TU containing the immediate early gene ICP0 (TU: LAT, RL1, RL2) was relatively consistent between strains and over time (**Fig 4B**), which was also reflected at the protein level in neurons, Vero, and HFF cells (**Fig 6**). Likewise, the TU encoding VP22 (TU: UL49A, UL49) was not statistically significantly different at the neuronal transcription level, and no differences in VP22 protein levels were observed between strains in infected neurons (**Fig 6A**). This was also true in infected Vero and HFF cells (**Fig 6B and 6C**). Together these data demonstrate our ability to detect strain-specific differences in gene transcription that correlate with differences in protein levels. These and the data above highlight the cell-type dependent differences in protein levels exhibited by different strains of HSV-1.

### Effects of HSV-1 F, KOS, and McKrae on adherens junction components

**Fig 7:**
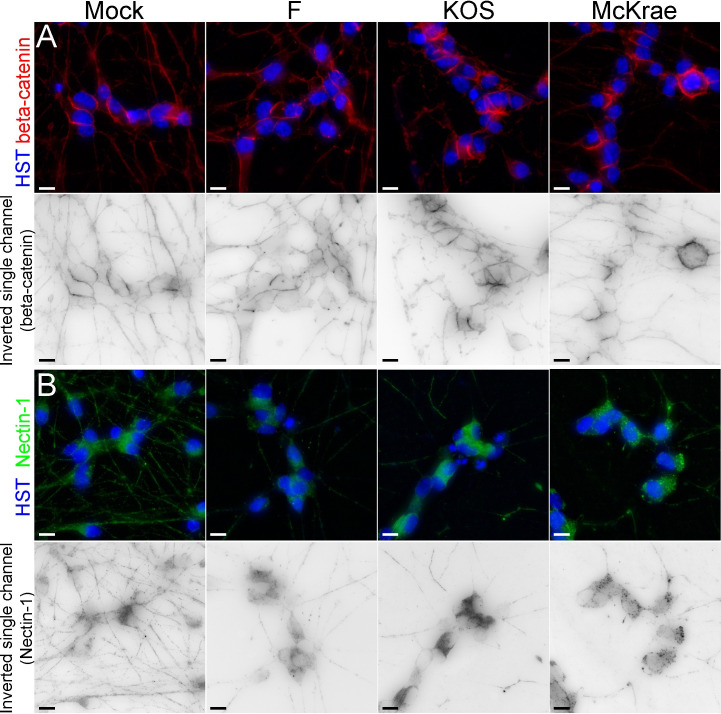
Neuronal adherens junction components reveal changes in levels following infection with HSV-1. **(A)** In mock-infected neurons, beta catenin is localized at cell borders, and along neurites. At 6 hours post infection with HSV-1, regardless of strain, beta-catenin displayed relatively similar beta-catenin immunofluorescence in neurons, although quantitation revealed higher beta-catenin levels for strain F than strain McKrae (**S6A Fig**). No differences in total beta catenin protein levels were observed by Western blot at 12 hpi (**S6B Fig**). **(B)** In mock-infected neurons, nectin-1 exhibits diffuse expression in neuronal cell bodies and along neurites. While the immunofluorescence of nectin-1 appeared to decrease after infection for all strains, particularly in the neurites, the overall quantitation of immunofluorescence revealed no changes in total fluorescence levels as compared with mock-infected neurons (**S6A Fig**). Western blot analysis of nectin-1 levels in infected neurons were inconclusive due to high background in neuronal cells (**S6B Fig**). For each overlay panel, a single-channel image of the neuronal marker (beta-catenin or nectin-1) is also included, where the fluorescence has been inverted for easier visualization of protein localization. (HST, Hoechst nuclear stain) Scale bar = 10 μm.

Remodeling of epithelial adherens junctions was one of the host neuronal pathways identified as differentially impacted by each strain of HSV-1 (**Fig 1B**), suggesting that disruption of cell-to-cell contacts may differ between virus strains. We therefore hypothesized that altered expression of gD (US6), gE (US8), and US9 between strains may differentially impact host adherens junction structure, and therefore virus spread. This hypothesis was supported by changes observed in neuronal morphology following infection (**Figs 5 and S4, S1–S4 Movies**), as well as transcriptional and protein level changes in gE (US8), and gD (US6) expression (**Figs 4C and 6A**). These viral glycoproteins have been shown to play key roles in interacting with adherens junctions during viral spread from cell-to-cell, and host nectin-1 and beta-catenin have both been implicated in these interactions with HSV-1 [69–75]. While all viral-infected groups demonstrated significantly elevated beta-catenin transcript expression versus mock-infected neurons, no differences in transcript expression were observed between specific HSV-1 strains (**Fig 1C**). To examine beta-catenin further, immunofluorescence assays were performed on neurons at 6 hpi with either HSV-1 F, KOS, or McKrae. In mock-infected neurons, beta-catenin protein was diffuse, with areas of concentration at cell soma borders, and beta-catenin immunofluorescence was evident along neuronal processes (**Fig 7A**). Infection with all HSV-1 strains resulted in relatively similar beta-catenin immunofluorescence in neurons (**Fig 7A**), although quantitation revealed higher beta-catenin levels for strain F infection than strain McKrae (**S6A Fig**). No differences in total beta-catenin protein levels were evident at 12 hpi when assessed by Western blot (**S6B Fig**).

While beta-catenin showed no differential transcript expression between strains, another adherens junction component and known HSV-1 entry mediator, nectin-1, was differentially expressed between virus strains in infected neurons (**Fig 1C**). To investigate potential strain-dependent differences in nectin-1 localization and production, neurons were infected with either F, KOS, or McKrae for 6 hours and then immunolabeled for nectin-1. In mock-infected neurons, nectin-1 protein was diffuse, as evidenced by dense immunostaining in the cell body and immunofluorescence throughout neuronal processes (**Fig 7B**). While the immunofluorescence of nectin-1 appeared to decrease in all infected neurons and particularly in the neurites (**Fig 7B**), the overall quantitation of immunofluorescence revealed no changes in total fluorescence levels as compared with mock-infected neurons (**S6A Fig**). Western blot analysis of nectin-1 levels in infected neurons were inconclusive due to high background in neuronal cells (**S6B Fig**). Together, these immunofluorescence data (at 6 hpi) and changes in neuronal morphology (at 12 and 24 hpi; see **Fig 5**) reflect the host transcriptional changes in pathways associated with neuronal adherens junctions, integrin signaling, and others (**Fig 1**).

## Discussion

It is clear that disease pathogenesis and prognosis are dependent on a combination of host- and virus-specific factors, especially in the case of HSV-1 infection. While many studies have elucidated host-cell-specific factors and viral factors that may contribute to HSV-1 disease and persistence, no studies have sought to simultaneously analyze both the host and viral transcriptomes during productive infection in different cell types and using different HSV-1 strains. Using this approach, the present study sought to reveal host- and virus-specific factors that may contribute to previously observed strain-dependent differences in HSV-1 neurovirulence *in vivo* [35, 40, 51]. To achieve this, we utilized an experimental model consisting of differentiated human SH-SY5Y neuroblastoma cells infected with one of three different strains of HSV-1 (F, KOS, and McKrae). Differences in virus and host transcription were analyzed in infected neurons over time and between virus strains (**S1 Fig**). This experimental approach allowed for the simultaneous assessment of both host and viral transcriptional changes during productive HSV-1 infection, providing important data on virus-host interactions in the specialized cell type of fully differentiated human neurons.

For both the viral and host transcriptomes, we found that virus strain used for infection had a significant impact on overall gene expression patterns. Both viral and host neuronal gene expression following infection with HSV-1 KOS were distinct from the patterns observed following infection with either F or McKrae (**Figs 1A and 3A**). This separation is corroborated by our observation of lower total viral protein levels in KOS-infected neurons (**Fig 6A**). Regardless of the HSV-1 strain used for infection, we saw changes in the transcriptional activation of a number of host pathways that regulate neuronal cell adhesion, migration, and cytoskeletal rearrangement (e.g., integrin, ILK, ephrin B, ephrin receptor signaling, and the regulation of epithelial adherens junctions; **Fig 1B**). Additionally, we observed the activation of host immune responses, including genes involved in pathways such as death receptor signaling and retinoic acid-mediated apoptosis signaling (**Fig 1B**). Broadly, these findings are in agreement with previous findings indicating the induction of host immune pathways with neuronal HSV-1 infection and latency, and the altered activation of neuronal cell adhesion and migration pathways [17, 19–21, 23, 24, 61].

Our data indicate that the three different strains of HSV-1 used in the current investigation cause host neurons to exhibit varying degrees of transcriptional responses for a number of these pathways. For example, many host pathways involved in signaling associated with cell adhesion, cytoskeletal reorganization, and cell migration demonstrate higher activation scores in KOS-infected neurons (**Fig 1B**). Differential activation of these pathways may alter cell adhesion and the number of cell contacts in a strain-specific manner, thereby impacting cell-to-cell spread of HSV-1. Analysis of neuronal morphology following infection (**Fig 5, S1–S4 Movies**), combined with these pathway data, suggest that there are strain-specific differences in the regulation of host adherens junction structure and cell-to-cell contacts. The HSV-1 protein gE localizes at adherens junctions, specifically with the host protein beta-catenin, and is hypothesized to utilize adherens junctions for cell-to-cell spread [69]. While the adherens junction protein beta-catenin demonstrated no overt strain-specific differences in protein levels, there appeared to be a subtle reduction in distal neurites after infection **(Figs 7 and S6)**. This observation echoes recent data from non-neuronal cells that demonstrated relocalization of beta-catenin to the nucleus and viral replication compartments after infection with HSV-1 strain 17, leading to altered host gene transcription and facilitating late viral gene expression [31]. We were unable to detect beta-catenin localization in the nucleus due to the inherent autofluorescence of these neurons. However, future investigations could use other methods, such as the expression of fluorescently-tagged beta catenin, to test whether nuclear relocalization of beta-catenin also occurs in HSV-1-infected neurons and if this process exhibits any strain-specific differences.

In addition to its interactions with beta-catenin, HSV-1 protein gD has been shown to bind to nectin-1, mediating viral entry, and potentially impacting adherens junction stability [70, 72, 73, 76]. Nectin-1 serves as an important entry receptor for HSV-1 [74, 77, 78]. Prior studies have demonstrated internalization and decreased expression of nectin-1 at cell junctions in non-neuronal cells following HSV-1 infection and/or after co-culture with gD expressing cells [72, 79]. It has been hypothesized that the viral protein gD disrupts nectin-1 homophilic intercellular trans-interactions at cell junctions, and may replace nectin-1 at the cell surface, potentially facilitating HSV-1 entry and cell-to-cell spread [72, 76]. Previous studies used different strains of HSV-1 KOS in their model systems [45, 46, 80]. We observed strain-dependent differences in nectin-1 transcript expression in infected neurons at 12 and 24 hpi (**Fig 1C**). At 6 hpi, we did not observe overt differences in nectin-1 protein levels between strains, although there appeared to be a relocalization of nectin-1 out of neurites (**Figs 7 and S6**). Due to the loss of adhesion to the substrate at later time points in infection (**Fig 5**), it was not possible to examine nectin-1 immunofluorescence at later timepoints in this neuronal model. Given the previously described interactions between gD and nectin-1, and the differential gene expression and protein levels of gD as observed here (**Figs 4 and 6A**), there may be additional effects on nectin-1 protein at later timepoints in infected neurons. This hypothesis remains to be tested. The current data in combination with prior works suggest that the lower transcript and protein levels of gE and gD in neurons may contribute to the observed attenuation of HSV-1 KOS *in vivo*, and also contribute to its slower rate of neuronal cell-to-cell spread both *in vitro* and *in vivo* [35, 40, 62]. These data may inform current studies using this popular laboratory model strain of HSV-1 [40, 44–46].

Viral strain divergence in the activation of host-neuron pathways, such as adherens junction remodeling and signaling, may be due to differences in the expression of viral genes between strains. Viral glycoproteins in particular (**Figs 3, 4 and 6**) may differentially impact the ability of the virus to enter and spread, and thus the magnitude or speed of host-cell responses. Observed differences in viral protein levels between strains were evident in both neurons and primary human fibroblasts to varying degrees, but were lacking in Vero cells (**Figs 6** and **S5**). It is well-known that Vero cells are defective in the production of interferon [81, 82]. This suggests that different strains of HSV-1 may have varying impacts on host cell immune responses, which could differ between cell types (e.g., neurons versus epithelial cells or fibroblasts). This hypothesis is supported by our data indicating that death receptor and retinoic acid-mediated apoptosis signaling demonstrates variable activation in neurons in a virus strain-specific manner, with KOS-infected neurons exhibiting the highest activation (**Fig 1B**). At present, it is unclear whether the differences in viral gene expression observed in this study (**Figs 3 and 4**) are more driven by viral genetics or by the strain-specific host response. However, it is likely that a complex interaction between virus strain-specific gene expression and host cell type-specific transcriptional responses contribute to the overall differences in pathogenesis and neurovirulence that have been previously observed for these strains *in vivo* [35, 40, 51]. Future studies should aim to investigate kinetic differences in viral gene expression and protein production between strains, in both epithelial and neuronal cell models.

HSV-1 strain comparisons using *in vivo* models have revealed that KOS is less neurovirulent when compared to other strains of HSV-1 [35, 40]. In prior analyses in human SH-SY5Y neurons, KOS exhibited slower replication than McKrae in a low-multiplicity of infection (MOI) assay of cell-to-cell spread (i.e. a multi-step growth curve) [62]. Our data using synchronous infection at a high dose (i.e. a single-step growth curve) demonstrate similar replication in differentiated SH-SY5Y neurons between strains F, KOS, and McKrae at early timepoints (0 to 12 hpi; **S3 Fig**). At 24 hpi and 48 hpi, strain F demonstrated statistically significant differences in titer. The similarity in replication at early timepoints suggests that inherent differences in viral replication are not driving the large transcriptomic differences observed between strains at 12 hpi (**Fig 1**). Rather it suggests that the early differences in viral and host transcriptomes which are evident at 12 hpi, lead to the later divergence in maximal titer achieved by each viral strain. The transcriptional differences observed here, in combination with additional inter-cellular signaling and immune responses that would normally occur in parallel during *in vivo* infection, may contribute to the previously observed divergence in overall neurovirulence of these strains in animal models [35, 40].

These and other circulating HSV-1 strains have numerous genetic differences [83–85], which have been proposed to impact observed phenotypic differences in HSV pathogenesis [86–88] and spread [89]. Many TUs containing HSV-1 glycoprotein transcripts (e.g., US5, US6, US7, US8, and UL44) demonstrated higher expression in F- and McKrae-infected neurons versus KOS-infected neurons (**Figs 3B and 4**). Conversely, the TU containing glycoprotein B (TU: UL27, UL28) exhibited higher expression in KOS-infected neurons at 24 hpi. Amino acid differences in HSV-1 glycoproteins can impact viral entry by modifying a strain’s ability to bind to cell entry receptors [52], an effect which can then be amplified over multiple rounds of cell to cell spread. Given the variable expression profiles of entry receptors between cell types [90], the observed differences in viral glycoprotein sequence, transcript expression, and protein levels between HSV-1 strains have the potential to impact virus spread in a cell-type-dependent manner. Alphaherpesviruses also exhibit genetic variability in short sequence repeats, which may impact transcriptional differences between strains [85, 91, 92], including of viral glycoproteins [91]. Future studies comparing these strains and their tandem repeat regions may provide key insights on strain-specific transcriptional regulation, and how the strain-specific expression level of key viral factors (e.g., glycoproteins) impacts viral spread and virulence.

The data presented here highlight the importance of using diverse strains of a given virus to probe neuron-virus interactions. Our study demonstrated that strain-specific differences in viral protein levels may become more pronounced in terminally differentiated neurons versus rapidly dividing cells such as Vero cells, which also lack an intact interferon response [81, 82] (**Fig 6)**. Neuron-specific differential expression of gE and gD, compounded with cell-type independent differences in US9 expression between HSV-1 strains, likely influence host adherens junction structure and signaling [44, 45, 47–50]. This may result in alterations in neuron-to-neuron spread capabilities between strains, which would ultimately contribute to differences in virus spread within the nervous system *in vivo*. Previous analysis of HSV-1 transcript expression in MRC5 epithelial cells and primary trigeminal ganglion neurons demonstrated differences in the accumulation of viral immediate early transcripts between these cell types [25]. Differences in viral transcript expression between cell types may be further exacerbated by the selection of virus strain(s) used for infection. These results highlight the need for future comparative studies that investigate inter-strain differences in gene expression and protein levels in different cell types, in order to understand how these differences impact host-cell responses.

Since SH-SY5Y neurons are derived from a neuroblastoma cell line, future studies may seek to include the comparison of strain-specific differences in viral transcription and host responses in different human neuronal cell types, such as iPSCs and LUHMES cells [60, 93, 94]. Characteristics of HSV-1 infection observed in epithelial cells (e.g., readthrough of host genes [26], or relocalization of beta-catenin and the requirement for late viral gene expression [31]) should be further investigated using neuronal cell models. A larger panel of HSV-1 strains could also be included in future studies, including diverse clinical strains. Our Western blot data indicate potential differences in the glycosylation of the viral glycoproteins gD and gE between HSV-1 strains, and the extent of this phenotype differs between cell types (**Fig 6**). Glycosylation of envelope proteins has been indicated as a determinant of virulence in flaviviruses [95, 96]. As such, future studies may also aim to characterize potential differences in HSV-1 virion protein glycosylation between strains and examine how this may differ by cell type as well. It is also important to note that many new and non-canonical ORFs have recently been detected in models of productive HSV-1 infection of non-neuronal cells [29, 34]. It would be beneficial for future studies utilizing long-read sequencing [30, 97] and/or mass spectrometry-based approaches [34] to explore whether or not these novel transcripts and/or translated products are also detected during neuronal infection, and whether differential expression is evident between HSV-1 strains.

## Methods

### Cell culture, infections, and virus strain characterization

Maintenance and differentiation of the human SH-SY5Y neuroblastoma cell line (ATCC, CLR-2266) was performed as previously described [57, 62], in an approach that is based on prior protocols [98, 99]. Briefly, undifferentiated SH-SY5Y cells cultured in 35 mm^2^ dishes were gradually serum-deprived over the course of 10 days by treating with Eagle’s minimal essential medium (EMEM, Sigma) supplemented with decreasing concentrations of heat-inactivated fetal bovine serum albumin (FBS) (Hyclone), 1X penicillin-streptomycin (Life Technologies-Gibco), 2 mM L-glutamine (Thermo Fischer Scientific-Hyclone) and 10 μM retinoic acid (Sigma). On Day 10, cells were passaged onto extracellular matrix-coated plates or coverslips (MaxGel, Sigma) and cultured in neuronal terminal differentiation media, containing Neurobasal (Life Technologies-Gibco), 1X B-27 (Thermo Fischer Scientific), 2 mM Glutamax (Life Technologies-Gibco), 1X penicillin/streptomycin, 1 M KCl, 2 mM dibutyryl cyclic AMP (Sigma), 50 ng/mL brain-derived neurotrophic factor (BDNF, Sigma), and 10 μM retinoic acid [57]. Cells were considered to be terminally differentiated on day 18 post initial plating. Vero African green monkey kidney cells (ATCC, CCL-81) and primary human foreskin fibroblasts (HFFs) were maintained in Dulbecco’s minimal essential medium (DMEM) supplemented with 10% FBS, 1X penicillin/streptomycin, and 2 mM L-glutamine. HFFs were derived from newborn male foreskin, and were kindly provided by Dr. Todd Ridky, University of Pennsylvania.

For all transcriptomic studies, terminally differentiated neurons were infected with either HSV-1 strain F [41], KOS_63D_ (provided by Richard Dix, referred to as KOS throughout, [35, 42]), or McKrae (obtained from James Hill, [43]). Strains F and KOS were originally isolated from the lips of patients with herpes labialis infection [35, 41, 42], while McKrae was isolated from the eye of a patient with HSV-1-associated keratitis [43]. All strains were passaged numerous times following isolation, and the exact genome sequences of each viral stock has been identified and published previously (F [84], KOS [45], and McKrae [100]). To ensure synchronous infection in light of the wide spacing of neurons relative to more densely plated fibroblast or epithelial cell lines, neurons were infected with 1.6e^7^ PFU per 35-mm^2^ dish (mock, n = 2; infected, n = 3-4/strain; each replicate was generated on the same day using the same passage neuronal culture). Purified virion preparations of each strain were used to infect neurons for the transcriptomic analysis. The virion purification protocol was based on prior protocols [101–103] and utilized a Nycodenz gradient instead of sucrose to improve virion integrity.

To determine the optimal infectious dose for synchronous infection of neuron cultures using purified virions, anti-HSV-1 immunofluorescence assays were performed. Fully differentiated neurons were infected with 1.2e^6^, 6e^6^, or 1.2e^7^ PFU of purified virions per 35-mm^2^ dish for 14 hours (n = 2 dishes per virus strain per dose). At 14 hpi, cells were rinsed with PBS, fixed with 3.2% paraformaldehyde/PBS for 10 minutes at room temperature, rinsed with PBS, and then permeabilized with 0.5% Triton X-100/3% BSA/PBS for 5 minutes. Once permeabilized, cells were blocked 0.1% Triton X-100/3% BSA/PBS for 30 minutes at room temperature, and then incubated with anti-HSV-1 primary antibody (DAKO, 1:100) in blocking buffer for 1 hour. Cells were washed twice with 3% BSA/PBS and then incubated with fluorescently-tagged anti-rabbit secondary antibody and Hoechst nuclear stain (anti-rabbit Alexa Fluor 633, 1:1000; Hoechst 1:1000). Following incubation with secondary antibody, cells were washed once with 3% BSA/PBS, twice with 0.1% Triton X-100/PBS, and then mounted with Aqua-Polymount (Polysciences) and stored at 4°C. Images were acquired using a Nikon Eclipse inverted epifluorescence microscope.

In order to test whether any differences in replication exist between F, KOS, and McKrae in neurons, a single-step growth curve was performed. Fully differentiated SH-SY5Y neurons grown in 35-mm^2^ dishes were infected with either F, KOS, or McKrae at a concentration of 1.6e^7^ PFU/dish (n = 4 dishes per strain per timepoint) and incubated for 1 hour at 37°C with rocking every 15 minutes. Following incubation, the inoculum was removed and replaced with fresh terminal differentiation media. Cells and media were then scraped and collected at 0, 6, 12, 24, and 48 hpi. Replicates from each timepoint were then titered on Vero cells. To determine strain- and time-dependent differences in replication, a 2-way ANOVA with interaction effects and post-hoc testing with Bonferroni multiple testing correction was performed.

All other experiments, including generation of SH-SY5Y, Vero, and HFF cell protein lysates, as well as fixed cells used for imaging, used an MOI of 10 for infection. Dilutions of viral inocula were prepared using neuronal terminal differentiation media for SH-SY5Y cells or DMEM supplemented with 2% FBS, 1X penicillin/streptomycin, and 2 mM L-glutamine for Vero and HFF cells. Mock-infected samples were treated with respective virus-free media alone. During infection, cells were incubated at 37°C for 1 hour with gentle rocking every 15 minutes. Media was then removed and replaced with fresh media.

### RNA isolation and sequencing

RNA was isolated from differentiated SH-SY5Y neuronal cells at 12 and 24 hpi using the manufacturer’s protocol for Trizol (Thermo Fisher Scientific), with minor adaptations for low input samples. To harvest neuronal samples, media was gently removed and Trizol added directly to each sample dish. Pipette-facilitated fluid motion was sufficient to dissolve the neuronal network and enable sample transfer to an Eppendorf tube. Chloroform (0.2 volumes) was added to the Trizol-sample mixture and vigorously mixed, before phase separation via centrifugation on a phase-lock gel tube. Linear polyacrylamide was added to the aqueous layer, along with isopropanol (0.5 volumes), for an overnight precipitation. After centrifugation, the pellet was washed with 70–75% ethanol twice, then dried, and resuspended in 10 mM Tris-Cl, pH 8.5.

Total RNA quality was assessed using a 2100 Bioanalyzer (Agilent Technologies), and RNA concentration was quantified by QuBit (average yield = ~1.1 μg per sample; Thermo Fisher Scientific). Library preparation was performed according to manufacturer’s instructions using 500 ng total RNA input (TruSeq RNA kit, Illumina, Protocol Part# 15026495 Rev. D). Quality and quantity of cDNA libraries was then assessed by QuBit and Bioanalyzer. Samples were normalized to a molarity of 10 nM and pooled for sequencing. Three independent sequencing runs were performed using 100 base-pair sequencing on Illumina HiSeq platforms at Princeton University (paired-end), or Penn State University (single-end). Raw RNAseq data have been deposited at the National Center for Biotechnology Information (NCBI) Sequence Read Archive (SRA) as BioProject number PRJNA593260.

### Host and virus transcriptomic analysis

RNA-sequencing data was assessed for quality using FastX-Toolkit and FASTQC to measure base quality scores and other relevant metrics. Reads from run one (paired-end sequencing) were trimmed of any adapters via FastX-Toolkit and low-quality bases (lower than Q30) were trimmed using a sliding window of 15 in Trimmomatic. Length based filtering was then applied, with any reads under thirty base pairs in length being discarded. Reads from runs two and three (single-end sequencing) were of higher quality, and did not require trimming.

To analyze host transcriptional changes in response to infection, reads were mapped to the host genome (*Homo sapiens* (release 37) reference sequence (GRCh37/hg19) using the HiSat aligner with default settings. Transcripts were assembled with StringTie using the GRCh37 reference to guide the assembly process and read counts were generated using an accompanying Python script (prepDE.py). Read counts were normalized in EdgeR [104] by sequencing depth and batch effect removal, and low expression transcripts (< -1 median log counts per million (CPM)) were removed. Normalized counts were then fitted to a negative binomial generalized log-linear model (GLM), using empirical Bayes tagwise dispersions to estimate the dispersion parameter for each transcript. Differentially expressed genes were identified using GLM likelihood ratio tests. Statistical significance was determined using a Benjimini Hochberg MTC (α = 0.05 threshold, false discovery rate (FDR) < 0.05).

Assessment of differential HSV-1 gene expression is challenging due to the limitations of short sequencing reads in resolving splicing and 3’ co-terminal transcripts [30]. We used bowtie2 [105] to align reads to viral transcripts to avoid spurious detection of splicing due to areas of high sequence similarity in the HSV-1 genome, and the nature of Illumina short read sequencing. Virus transcripts were counted once per transcriptional unit (TU), such that non-overlapping gene transcripts were counted uniquely, and overlapping transcripts were counted as one unit (**Fig 2**, **S4 Table**). Virus TU data were normalized in EdgeR [104] for sequencing library size, sequencing run batch effects, and low expression transcripts. Normalized data was analyzed for differential gene expression using a GLM and quasi-likelihood F-tests. Statistical significance was determined using a Benjimini Hochberg MTC at an α = 0.05 threshold, and FDR < 0.05. Plots for both human and viral transcriptome data were generated using the ggplot2, ggfortify, ggbiplot, ggrepel, pheatmap, and EnhancedVolcano packages [106–111].

To determine pathways of host genes that are differentially regulated following HSV-1 infection, host-specific genes identified as differentially expressed following infection with either F, KOS, or McKrae at 12 hpi (p<0.05, Benjimini Hochberg MTC) were analyzed using Ingenuity Pathway Analysis (IPA) software (Qiagen). We then performed a Comparison Analysis within IPA to determine host pathways that may be differentially impacted by each HSV-1 strain.

### Protein isolation and Western blotting

Neuronal protein lysates were isolated at 12 hpi as previously described (n = 4 independent neuronal cultures) [62]. Briefly, neurons were rinsed twice with 1X PBS, lysed in Radio Immunoprecipitation Assay (RIPA) buffer (Sigma Aldrich) supplemented with Pierce protease and phosphatase inhibitor (PPi) mini tablets (Thermo Scientific), scraped, and pooled into 1.5 mL centrifuge tubes. To ensure minimal loss of protein during media removal and washes, all media and PBS were removed, combined, and centrifuged at 1,000 x g for 2 minutes to pellet any cells that had lifted off of dishes during processing. Pellets were then washed twice with PBS, resuspended in RIPA + PPi, and combined with scraped cells. Vero protein lysates at 6 hpi were prepared by rinsing twice with 1X PBS, lysing in RIPA buffer supplemented with protease and phosphatase inhibitor tablets, and then scraping into 1.5 mL centrifuge tubes. All lysates were sonicated (80% amplitude, 10 seconds on/off, Q500 ultrasonic processor), rocked for 15 minutes at 4°C, and then centrifuged at 4°C at 12,500 x g for 10 minutes. Soluble protein present in the supernatant was removed and quantified using a Bicinchoninic acid (BCA) assay (Thermo Scientific) and a Nanodrop 2000c spectrophotometer.

Host and viral protein levels were assessed by Western blot as previously described [62]. Equal concentrations of protein were loaded and separated by sodium dodecyl sulfate polyacrylamide gel electrophoresis (SDS-PAGE) (Miniprotean; Bio-Rad), and then transferred to nitrocellulose membranes (Amersham GE Healthcare) using a Trans Blot SD semi-dry electrophoresis transfer cell (Bio-Rad). Membranes were blocked with 5% non-fat dry milk in wash buffer (1 M Tris (pH 7.4), 154 mM NaCl, 0.2% Tween 20) overnight at 4°C with gentle rocking. Blocked membranes were then incubated with primary antibody (see **S5 Table**) diluted in blocking buffer for 2 hours at room temperature. Membranes were then washed 3 times, incubated with species-specific secondary antibody diluted in blocking buffer for 1 hour at room temperature, washed again, and developed using either enhanced chemiluminescence substrate or SuperSignal West Dura substrate (Thermo Scientific). Generation of pixel intensity profiles of representative lanes and band volume quantitation were performed using ImageQuant 8.1 (GE Healthcare), with image rectangle background subtraction applied to all images.

### Immunofluorescence of fixed cells

Immunofluorescence assays were performed as previously described (n = 3 independent neuronal cultures, 2 coverslips per condition) [62, 112]. Partially differentiated SH-SY5Y cells were plated onto MaxGel (Sigma) coated coverslips on day 10 of differentiation and maintained until terminal differentiation on day 18 [57]. Following terminal differentiation, neurons were counted and then infected with KOS, F, or McKrae at an MOI of 10 for 6 hours. We were unable to perform immunofluorescence assays on fully differentiated SH-SY5Y cells at 12 hpi, since most infected neurons lose their contact adhesion at this stage of infection. At 6 hpi, coverslips were rinsed twice with 1X PBS, fixed with 4% paraformaldehyde/PBS, washed with PBS, and then permeabilized with 0.1% Triton X-100/PBS for 10 minutes. Cells were then blocked with 10% goat serum diluted in PBS for 1 hour at room temperature and incubated with primary antibody (see **S5 Table**) diluted in the same blocking buffer overnight at 4°C in a humid chamber. Coverslips were washed in PBS, and incubated in species-specific fluorescence-labeled F(ab’)_2_ fragment secondary antibody (Jackson ImmunoResearch) and Hoechst nuclear stain (HST; 1:10,000) for 1.5 hours at room temperature in a light-protected humid chamber. Cells were then washed in PBS and mounted onto glass slides using ProLong Gold antifade mounting medium (Thermo Fischer Scientific) and allowed to dry overnight. Images were acquired using a Nikon Ti Eclipse inverted epifluorescence microscope with equivalent laser settings applied to all coverslips within a comparison. Image z-stacks were imported into Fiji (ImageJ) where each sample was visualized as a maximum projection and background subtraction was applied (rolling ball radius = 100 pixels). Image quantitation was performed in Fiji (ImageJ), where each image was visualized as a sum projection, background subtracted, and the mean gray value was assessed. For each group, 5 to 14 fields of view were analyzed. Processed images were then imported into Adobe PhotoShop where brightness and contrast was adjusted equally across images within a comparison.

### Live-cell imaging

Acquisition of time-lapse videos was performed as previously described [62]. Briefly, fully differentiated SH-SY5Y neuroblastoma cells were infected with viral inocula diluted in neuronal terminal differentiation media for 1 hour with gentle rocking every 15 minutes. Mock-infected neurons were treated with neuronal terminal differentiation media only. Following the 1 hour incubation, inocula was removed and 1 mL fresh warmed neuronal terminal differentiation media was added to each 35 mm^2^ dish. Brightfield images were acquired of mock-, F-, KOS-, and McKrae-infected neurons at 20X magnification every hour for 24 hours using a Nikon Ti Eclipse microscope equipped with a stage-top incubator (Tokai Hit). For each sample, three image series per well were acquired (Mock, n = 2 wells; F, KOS, McKrae, n = 3 wells). Time-lapse data were imported into Fiji, brightness and contrast of brightfield images were adjusted, and movies were exported at 1 frame/second.

### Scanning electron microscopy (SEM)

Neurons were differentiated and passaged onto MaxGel-coated coverslips as described above (n = 2 independent neuronal cultures, 2 coverslips each). Following terminal differentiation, neurons were either mock-infected with media containing no virus or infected with HSV-1 McKrae at an MOI of 10 for 6 hours. Due to insufficient cell adhesion to withstand the SEM sample preparation and imaging, it was not possible to collect SEM data at 12 hpi. Coverslips were rinsed twice with 1XPBS and then fixed with 2.5% glutaraldehyde in 0.1M sodium phosphate buffer at room temperature for 30 minutes. Neurons were washed 3 times with 0.1M sodium phosphate buffer, and then dehydrated in a series of ethanol washes (25%, 50%, 70%, 85%, and 95% one time each for 5 minutes, and then 100% ethanol 3 times). Dehydrated cells were prepared for EM by critical point drying (Leica EM CPD300, Leica, Wetzlar, Germany) and sputter-coating with gold (Denton). Neuronal cells were imaged using a Zeiss Sigma VP-FESEM (Zeiss, Thornwood, NY).

## Supporting information

S1 Fig Experimental design to interrogate host and viral transcription in HSV-1-infected neurons. The goal of the present study was to detect both the virus- and host-specific differences in response to infection, which may contribute to previously observed differences between HSV-1 strains in terms of their neurovirulence *in vivo* [35, 40, 51]. To achieve this, terminally differentiated human SH-SY5Y neuroblastoma cells were mock-infected or infected with either F, KOS, or McKrae. Relative neurovirulence of HSV-1 strains *in vivo* is indicated by the red bar, increasing from strains KOS and F to the highly virulent strain McKrae. While Dix *et al* found KOS and F to be similar in neurovirulence *in vivo*, strain KOS has since been sequenced and revealed to lack US9, which plays a role in neuronal transport [35, 44–50]. Total RNA was isolated at 12 hpi (infection midpoint) and 24 hpi (peak virus production). RNA was sequenced using Illumina technology to detect differences in viral and host gene transcription between viral strains and over time. Following identification of potential proteins and pathways of interest, targeted Western blots and immunofluorescence assays were performed to confirm transcriptomic findings.
(TIF)

S2 Fig Determination of infectious dose required for synchronous neuronal HSV-1 infection. Immunofluorescence assays were used to determine the optimal infectious dose of purified virions of HSV-1 F, KOS, and McKrae that is required to produce synchronous infection in differentiated SH-SY5Y neurons. Neurons were infected at an infectious dose of 1.2e^6^, 6e^6^, or 1.2e^7^ PFU/dish and incubated for 14 hours. Neurons were then fixed, permeabilized, and probed with anti-HSV-1 antibody (**S5 Table**), and the presence of HSV-1 in neurons was assessed by immunofluorescence. At the lower doses used here, neuronal cell bodies lacking HSV-1 immunoreactivity were evident in F- and KOS-infected dishes. At an infectious dose of 1.2e^7^, all neuronal cell bodies, regardless of HSV-1 strain used, were infected. HSV-1, red; nuclei, blue; n = 2 per strain. Scale bar represents 100 μm.
(TIF)

S3 Fig Single-step growth curve analysis demonstrates few differences in viral replication between strains in differentiated SH-SY5Y neurons. To determine whether HSV-1 strains F, KOS, and McKrae differ in their ability to replicate in neurons, a single-step growth curve was performed. Differentiated SH-SY5Y neurons were infected with 1.6e^7^ PFU/dish of F, KOS, or McKrae. Neurons were harvested at 0, 6, 12, 24, and 48 hpi and titered on Vero cells. While there was no significant main effect of virus strain on titer, time post-infection did impact viral titer (p<0.0001) and a significant interaction exists between HSV-1 strain and time post-infection (p<0.0001). Data are shown as average titer ± standard deviation (n = 4 dishes per strain per timepoint). A two-way ANOVA with interaction effects and post-hoc testing was performed followed by a Bonferroni multiple testing correction. *p = 0.009 F vs McKrae, **p<0.001 F vs McKrae, ##p<0.001 F vs KOS.
(TIF)

S4 Fig Inverted brightfield images of mock- and HSV-1-infected neurons. Different HSV-1 strains are depicted in columns, and 12 vs 24 hpi in rows. Images from **Fig 5A** were inverted and brightness and contrast were equally adjusted to better visualize changes in neurite adhesion and fasciculation following infection. The neurons appear to have decreased attachment or adhesion to the substrate at both 12 and 24 hpi, with increased bundling or fasciculation of neurites. Scale bars = 100 μm.
(TIF)

S5 Fig Quantitation of Western blot data demonstrating cell type- and strain-specificity of viral protein levels. Replicate Western blots probed for gD, gE, and total viral protein (HSV-1) were quantitated. These include the representative Western blot images shown in Fig 6, as well as additional replicate blots (n = 2–4 experiments per strain per cell type, each prepared and immunoblotted independently). In neurons and primary human fibroblasts (HFFs), the levels of gD, gE, and total HSV-1 protein were lower in KOS-infected cells than for either of the other two strains. This effect was most noticeable for gD. In Vero cells, the levels of these viral proteins were roughly equivalent across all three HSV-1 strains. Data were normalized to the average band volume for F-infected cells and are shown as the average band volume ± standard error.
(TIF)

S6 Fig Immunofluorescence quantitation and Western blots of neuronal adherens junction components following infection with HSV-1. **A**). The total fluorescence of beta-catenin and nectin-1 immunostaining of mock-infected or HSV-1-infected SH-SY5Y neurons was quantitated in Fiji. Beta-catenin immunofluorescence was significantly different after infection by strain F vs McKrae by one-way ANOVA (*p < 0.05). These data are plotted as the mean gray value ± standard error (n = 5–14 fields of view per group). These data include the representative immunofluorescence images shown in Fig 7, as well as additional fields of view. **B**) At 12 hpi, mock-infected SH-SY5Y neurons and neurons infected with either HSV-1 F, KOS, or McKrae were probed for levels of beta-catenin and nectin-1 protein. Consistent with immunofluorescence assays, no differences in beta-catenin levels were observable between groups. Nectin-1 levels appeared marginally higher in neurons infected with KOS versus mock-, F-, or McKrae-infected neurons across multiple replicates, but in all cases the level of background was too high for quantitation. Neuronal lysates shown here are from the same biological replicate as shown in the panel of Western blots in **Fig 6A**, and GAPDH (depicted in **Fig 6A**) was used as the loading control. The GAPDH loading control is not repeated here, to avoid image duplication.
(TIF)

S1 Table Number of reads mapping to neuronal and HSV-1 transcriptomes for RNA-sequencing data. (XLSX)

S2 Table This file lists the logCPM values for all host transcripts (spreadsheet tab #1), and those host transcripts identified as differentially expressed (versus mock-infected neurons) at 12 hpi with HSV-1 strain F, KOS, or McKrae (spreadsheet tabs #2–4). The data in tabs #2–4 served as the input for pathway analysis (Ingenuity Pathway Analysis; see Methods for details).
(ZIP)

S3 Table This file lists the canonical host pathways identified as differentially impacted by HSV-1 strain (spreadsheet tab #1), along with three tables of strain-specific data on the host neuronal pathways significantly regulated by HSV-1 infection at 12 hpi with strain F, KOS, or McKrae (spreadsheet tabs #2–4). Table abbreviations: FDR, false discovery rate-adjusted P value; LR, likelihood ratio; logCPM, log counts per million; logFC, log fold-change.
(XLSX)

S4 Table Virus transcriptional units (TUs) and gene functions (spreadsheet tab #1) and the log_2_ fold-change (LogFC) of statistically significant viral TU’s; (p-value<0.05, FDR<0.05) (spreadsheet tab #2). (XLSX)

S5 Table List of antibodies used in this manuscript along with dilutions, catalog numbers, and sources. (XLSX)

S1 Movie Mock-infected neurons. One image was acquired every hour for 24 hours.
(AVI)

S2 Movie F-infected neurons. One image was acquired every hour for 24 hours.
(AVI)

S3 Movie KOS-infected neurons. One image was acquired every hour for 24 hours.
(AVI)

S4 Movie McKrae-infected neurons. One image was acquired every hour for 24 hours.
(AVI)
