# Supplementary figures and images for: Viral infection of human neurons triggers strain-specific differences in host neuronal and viral transcriptomes
Source: PLoS Pathog. 2021 Mar 22;17(3):e1009441. doi: 10.1371/journal.ppat.1009441 (PMC8016332; doi:10.1371/journal.ppat.1009441)

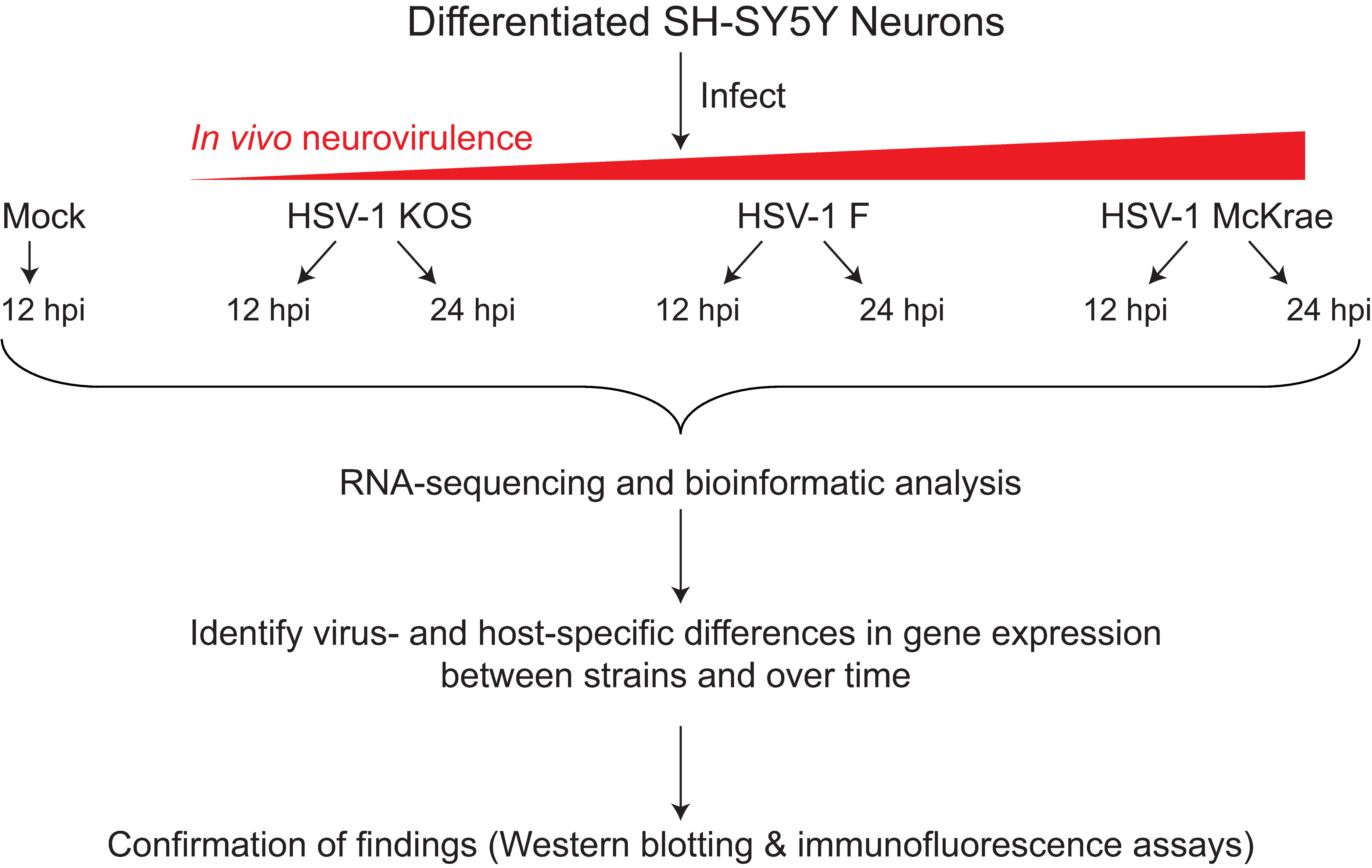

Supplement: S1 Fig — The goal of the present study was to detect both the virus- and host-specific differences in response to infection, which may contribute to previously observed differences between HSV-1 strains in terms of their neurovirulence in vivo [35, 40, 51]. To achieve this, terminally differentiated human SH-SY5Y neuroblastoma cells were mock-infected or infected with either F, KOS, or McKrae. Relative neurovirulence of HSV-1 strains in vivo is indicated by the red bar, increasing from strains KOS and F to the highly virulent strain McKrae. While Dix et al found KOS and F to be similar in neurovirulence in vivo, strain KOS has since been sequenced and revealed to lack US9, which plays a role in neuronal transport [35, 44–50]. Total RNA was isolated at 12 hpi (infection midpoint) and 24 hpi (peak virus production). RNA was sequenced using Illumina technology to detect differences in viral and host gene transcription between viral strains and over time. Following identification of potential proteins and pathways of interest, targeted Western blots and immunofluorescence assays were performed to confirm transcriptomic findings. (TIF) [file ppat.1009441.s001.tif]

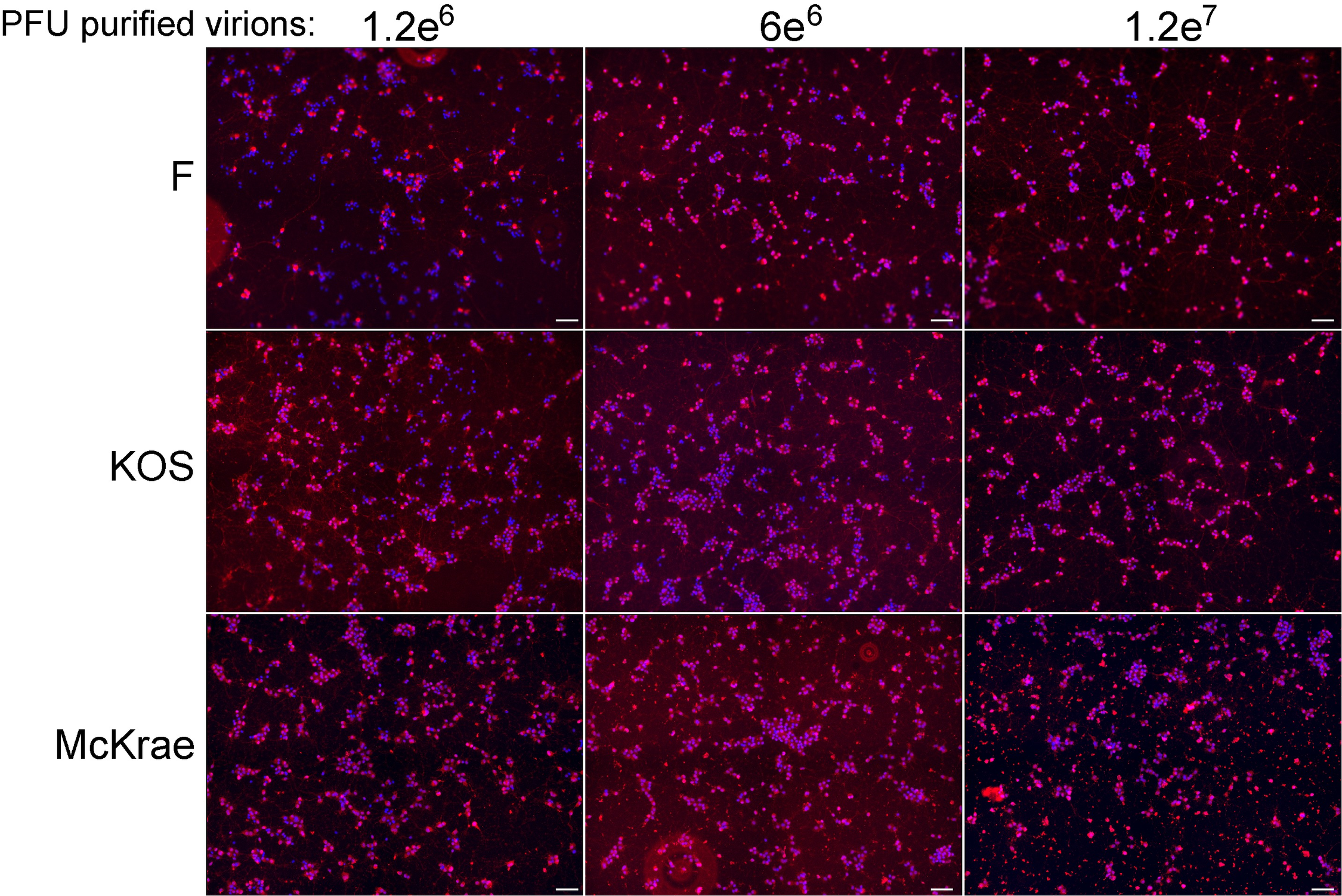

Supplement: S2 Fig — Immunofluorescence assays were used to determine the optimal infectious dose of purified virions of HSV-1 F, KOS, and McKrae that is required to produce synchronous infection in differentiated SH-SY5Y neurons. Neurons were infected at an infectious dose of 1.2e6, 6e6, or 1.2e7 PFU/dish and incubated for 14 hours. Neurons were then fixed, permeabilized, and probed with anti-HSV-1 antibody (S5 Table), and the presence of HSV-1 in neurons was assessed by immunofluorescence. At the lower doses used here, neuronal cell bodies lacking HSV-1 immunoreactivity were evident in F- and KOS-infected dishes. At an infectious dose of 1.2e7, all neuronal cell bodies, regardless of HSV-1 strain used, were infected. HSV-1, red; nuclei, blue; n = 2 per strain. Scale bar represents 100 μm. (TIF) [file ppat.1009441.s002.tif]

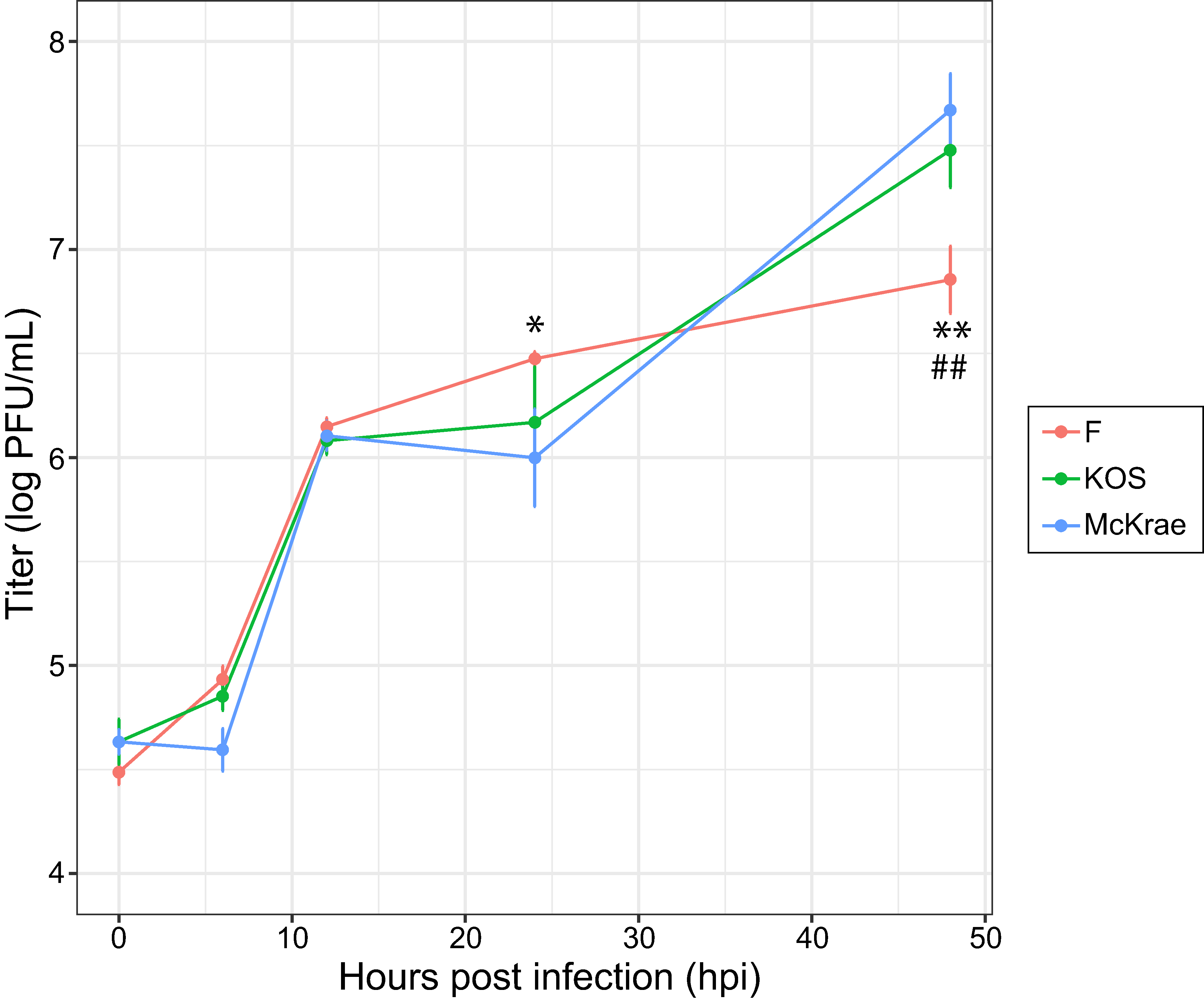

Supplement: S3 Fig — To determine whether HSV-1 strains F, KOS, and McKrae differ in their ability to replicate in neurons, a single-step growth curve was performed. Differentiated SH-SY5Y neurons were infected with 1.6e7 PFU/dish of F, KOS, or McKrae. Neurons were harvested at 0, 6, 12, 24, and 48 hpi and titered on Vero cells. While there was no significant main effect of virus strain on titer, time post-infection did impact viral titer (p<0.0001) and a significant interaction exists between HSV-1 strain and time post-infection (p<0.0001). Data are shown as average titer ± standard deviation (n = 4 dishes per strain per timepoint). A two-way ANOVA with interaction effects and post-hoc testing was performed followed by a Bonferroni multiple testing correction. *p = 0.009 F vs McKrae, **p<0.001 F vs McKrae, ##p<0.001 F vs KOS. (TIF) [file ppat.1009441.s003.tif]

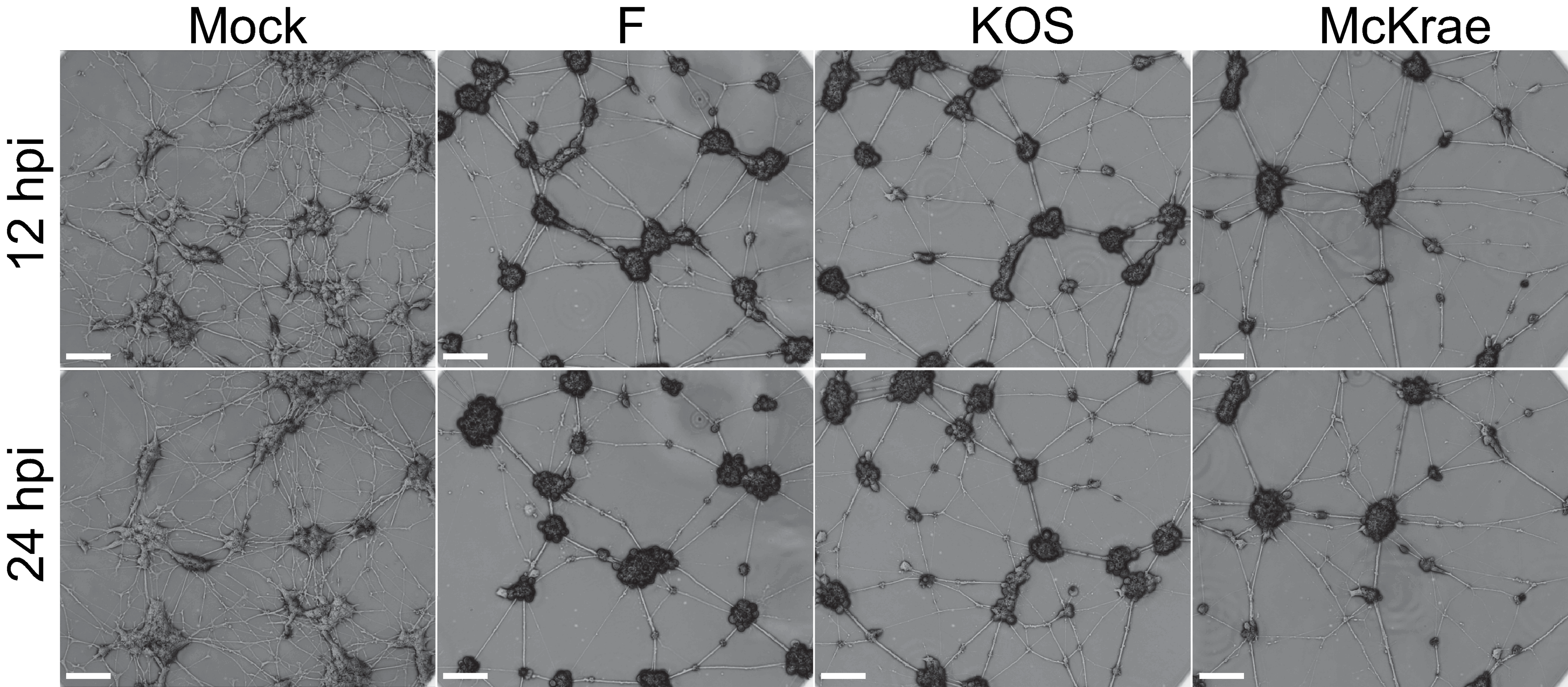

Supplement: S4 Fig — Different HSV-1 strains are depicted in columns, and 12 vs 24 hpi in rows. Images from Fig 5A were inverted and brightness and contrast were equally adjusted to better visualize changes in neurite adhesion and fasciculation following infection. The neurons appear to have decreased attachment or adhesion to the substrate at both 12 and 24 hpi, with increased bundling or fasciculation of neurites. Scale bars = 100 μm. (TIF) [file ppat.1009441.s004.tif]

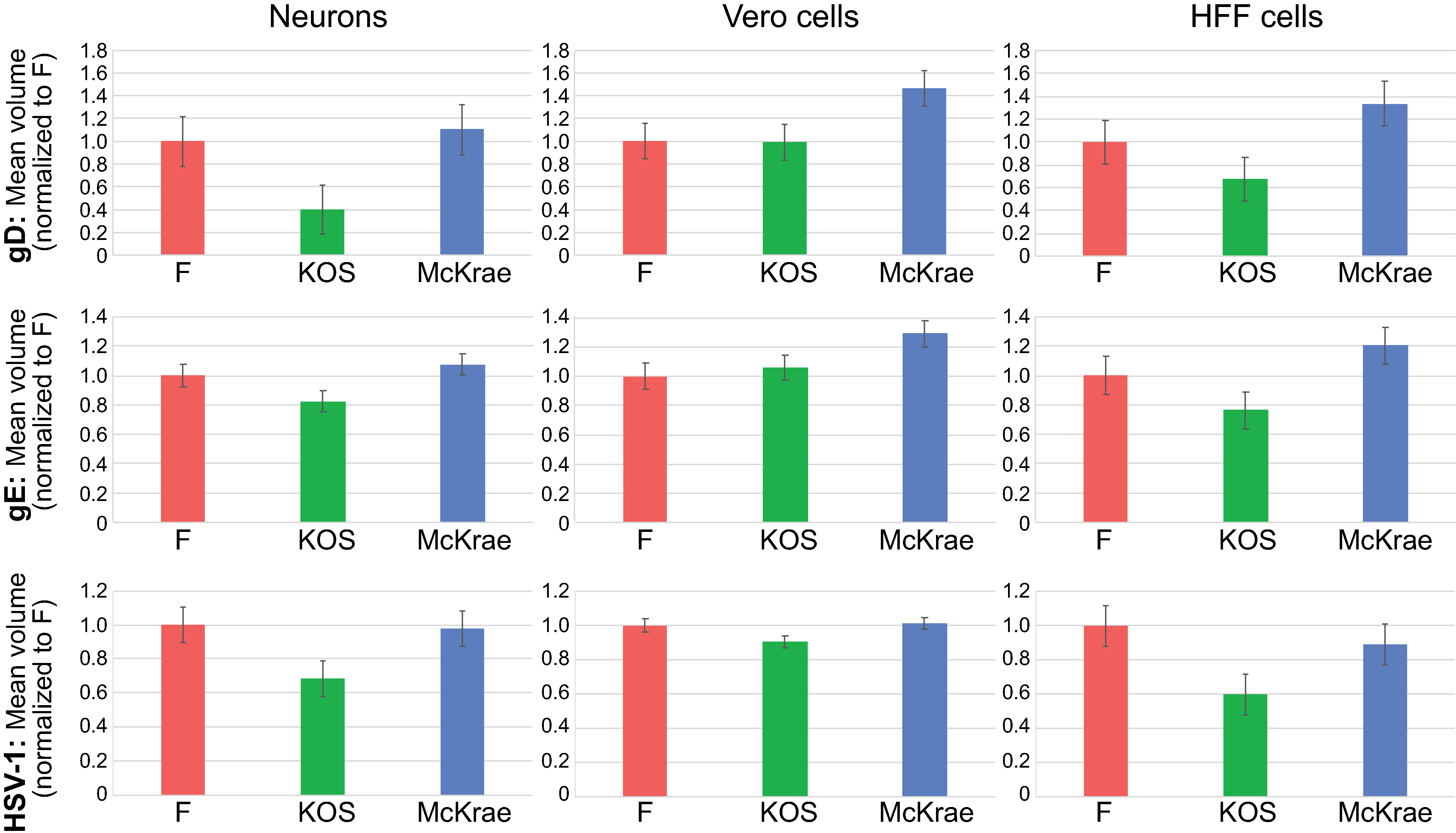

Supplement: S5 Fig — Replicate Western blots probed for gD, gE, and total viral protein (HSV-1) were quantitated. These include the representative Western blot images shown in Fig 6, as well as additional replicate blots (n = 2–4 experiments per strain per cell type, each prepared and immunoblotted independently). In neurons and primary human fibroblasts (HFFs), the levels of gD, gE, and total HSV-1 protein were lower in KOS-infected cells than for either of the other two strains. This effect was most noticeable for gD. In Vero cells, the levels of these viral proteins were roughly equivalent across all three HSV-1 strains. Data were normalized to the average band volume for F-infected cells and are shown as the average band volume ± standard error. (TIF) [file ppat.1009441.s005.tif]

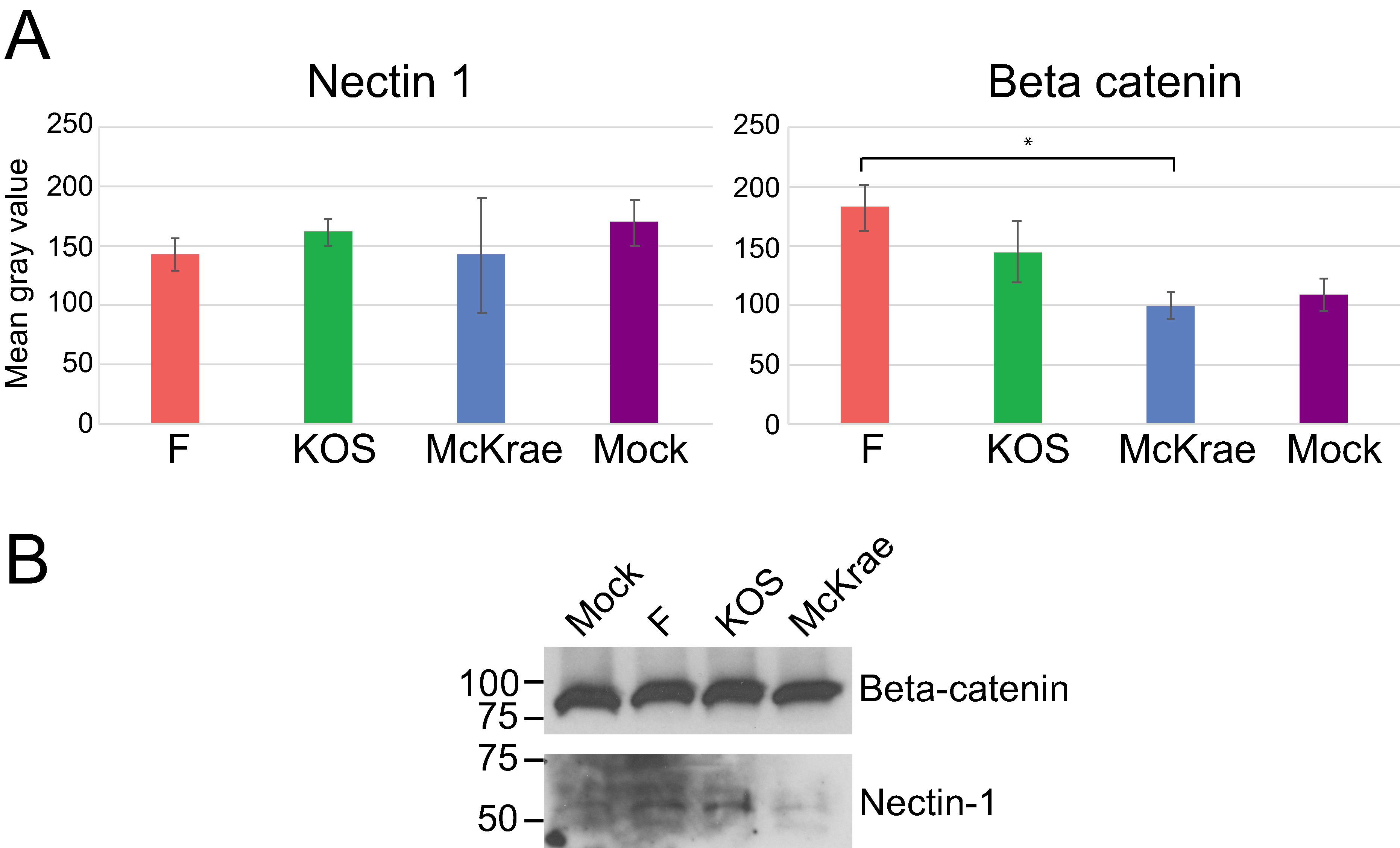

Supplement: S6 Fig — A). The total fluorescence of beta-catenin and nectin-1 immunostaining of mock-infected or HSV-1-infected SH-SY5Y neurons was quantitated in Fiji. Beta-catenin immunofluorescence was significantly different after infection by strain F vs McKrae by one-way ANOVA (*p < 0.05). These data are plotted as the mean gray value ± standard error (n = 5–14 fields of view per group). These data include the representative immunofluorescence images shown in Fig 7, as well as additional fields of view. B) At 12 hpi, mock-infected SH-SY5Y neurons and neurons infected with either HSV-1 F, KOS, or McKrae were probed for levels of beta-catenin and nectin-1 protein. Consistent with immunofluorescence assays, no differences in beta-catenin levels were observable between groups. Nectin-1 levels appeared marginally higher in neurons infected with KOS versus mock-, F-, or McKrae-infected neurons across multiple replicates, but in all cases the level of background was too high for quantitation. Neuronal lysates shown here are from the same biological replicate as shown in the panel of Western blots in Fig 6A, and GAPDH (depicted in Fig 6A) was used as the loading control. The GAPDH loading control is not repeated here, to avoid image duplication. (TIF) [file ppat.1009441.s006.tif]
